# Structure of human RNA polymerase III

**DOI:** 10.1038/s41467-020-20262-5

**Published:** 2020-12-17

**Authors:** Ewan Phillip Ramsay, Guillermo Abascal-Palacios, Julia L. Daiß, Helen King, Jerome Gouge, Michael Pilsl, Fabienne Beuron, Edward Morris, Philip Gunkel, Christoph Engel, Alessandro Vannini

**Affiliations:** 1 Division of Structural Biology, The Institute of Cancer Research, London, SW7 3RP UK; 2 Regensburg Center for Biochemistry, University of Regensburg, 93053 Regensburg, Germany; 3 Max Planck Institute for Biophysical Chemistry, Research Group Nuclear Architecture, 37077 Göttingen, Germany; 4 Fondazione Human Technopole, Structural Biology Research Centre, 20157 Milan, Italy

**Keywords:** CRISPR-Cas9 genome editing, Transcription, Cryoelectron microscopy, SAXS, X-ray crystallography

## Abstract

In eukaryotes, RNA Polymerase (Pol) III is specialized for the transcription of tRNAs and other short, untranslated RNAs. Pol III is a determinant of cellular growth and lifespan across eukaryotes. Upregulation of Pol III transcription is observed in cancer and causative Pol III mutations have been described in neurodevelopmental disorders and hypersensitivity to viral infection. Here, we report a cryo-EM reconstruction at 4.0 Å of human Pol III, allowing mapping and rationalization of reported genetic mutations. Mutations causing neurodevelopmental defects cluster in hotspots affecting Pol III stability and/or biogenesis, whereas mutations affecting viral sensing are located in proximity to DNA binding regions, suggesting an impairment of Pol III cytosolic viral DNA-sensing. Integrating x-ray crystallography and SAXS, we also describe the structure of the higher eukaryote specific RPC5 C-terminal extension. Surprisingly, experiments in living cells highlight a role for this module in the assembly and stability of human Pol III.

## Abstract (web summary)

The eukaryotic RNA Polymerase III transcribes tRNAs, some ribosomal and spliceosomal RNAs. Here, the authors resolve a cryo-EM structure of human RNA Polymerase III in its apo form and complemented it with crystal structures and SAXS analysis of RPC5, revealing insights into the molecular mechanisms of Pol III transcription.

## Introduction

Transcription of the eukaryotic genome is mediated by three highly specialized nuclear RNA polymerase (Pol) enzymes. Pol III transcribes short untranslated RNAs, which are essential for cellular functions, such as the entire pool of transfer RNAs, the precursor of the 5S ribosomal RNA and the U6 spliceosomal RNA^1^.

Pol III is a multi-subunit complex composed of 17 subunits. A central ten-subunit core, which harbours the catalytic site and a peripheral heterodimeric stalk that are structurally conserved among the three eukaryotic Pols. The TFIIF-like RPC4/5 and the TFIIE-like RPC3/6/7 subcomplexes are Pol III specific and can be regarded as built-in general transcription factors that play a fundamental role in Pol III transcription initiation and termination^2–4^.

Across the eukaryotic kingdom, Pol III displays a high degree of conservation both in terms of subunit composition and sequence homology of the individual components. A notable exception is the subunit RPC5, which in metazoans encompasses a long C-terminal extension (RPC5EXT, ~450 residues long), whose function is currently unknown.

Pol III activity is highly regulated in a cell cycle and cell-type-dependent manner^5^, and is a determinant of lifespan in eukaryotes^6^. In recent years, a large number of disease-causing mutations have been assigned to Pol III subunits, with a particular incidence of allele variants that strongly affect the correct development of the central nervous system (CNS), resulting in severe neurodegenerative diseases^7–14^. Furthermore, causative Pol III mutations have also been described in patients affected by hypersensitivity to viral infection^15,16^.

To date, yeast Pol III has been extensively structurally and functionally characterized, while its human counterpart has been left relatively untouched, due to the inherent technical challenges in obtaining yields amenable for structural biology. However, understanding the specific influence of pathological mutations and the role of regulatory elements unique to the human enzyme relies on such structural information. Here, we report the cryogenic electron microscopy (cryo-EM) reconstruction of human Pol III. We further study the enzymes’ complete architecture using a structural biology hybrid approach integrating two crystal structures of the human RPC5 C-terminal extension, as well as SAXS data and molecular modelling. Results of our comparative structural analysis rationalize the effect of pathological mutations and yield unexpected insights into Pol III regulation.

## Results

### Purification of human RNA Pol III

**Fig. 1:**
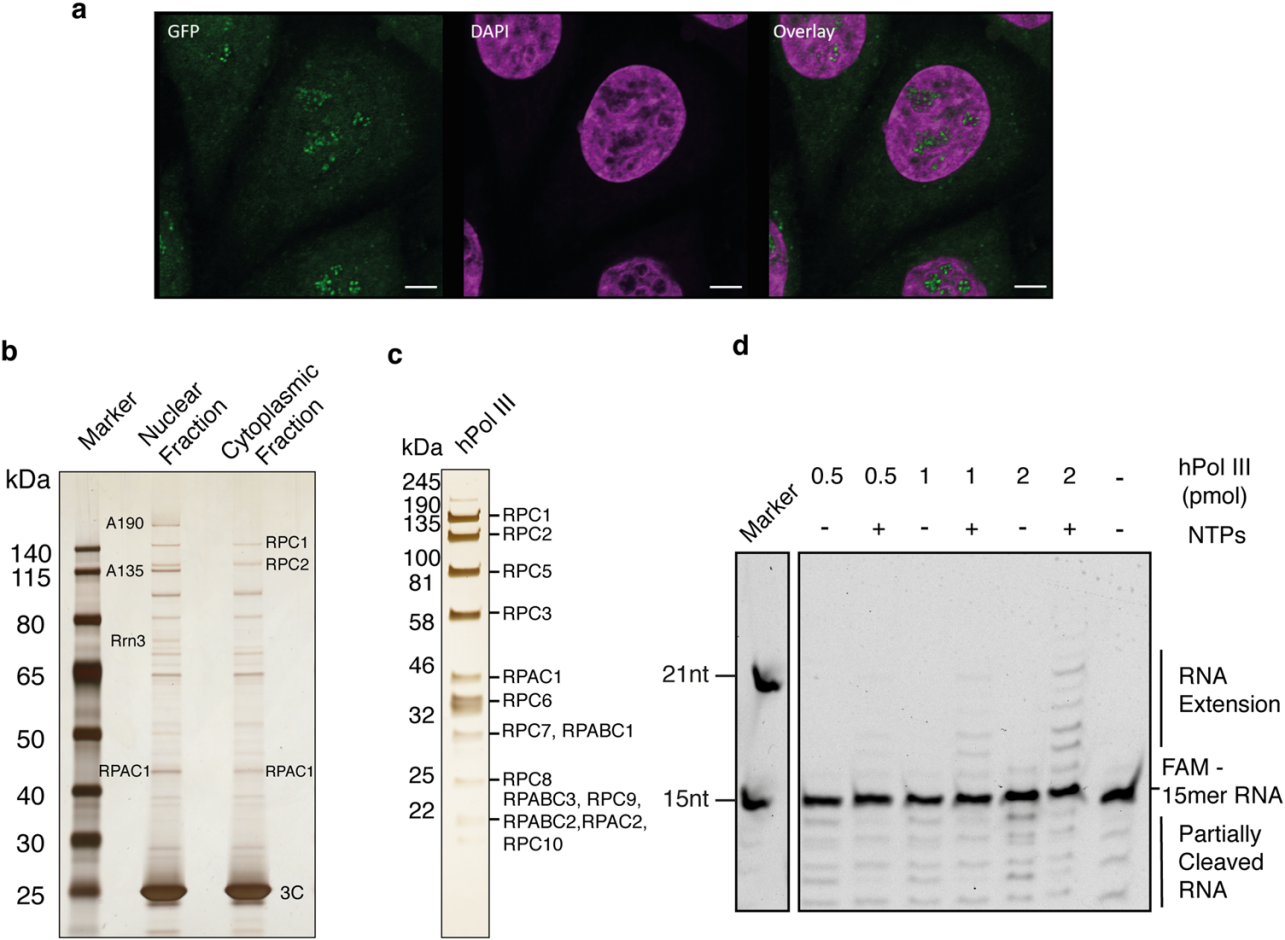
Purification of GFP-tagged endogenous human RNA polymerase III. **a** Confocal imaging of modified HeLa cell line expressing homozygous sfGFP-tagged POLR1C gene. Endogenous GFP signal, representing Pol I and III (green), DAPI staining (magenta) and overlay of both channels are shown. Scale bar: 5 µm. Shown is a representative image of four independent experiments. **b** Affinity-purified human RNA Pol III from HeLa nuclear and cytoplasmic fractions. Confirmed RNA Pol subunits are labelled. Shown is a representative image of two separate experiments. **c** Purified human RNA Pol III (hPol III) from large-scale whole-cell lysate with Pol III subunits marked. Shown is an excised lane of a representative image of three separate purifications. **d** RNA extension assay of fluorescently labelled FAM-15mer RNA primer by purified human Pol III. Marked is Pol III-mediated primer extension. The molecular marker displays expected sizes for 15 and 21 nucleotides (nt). Displayed is a representative image of seven independent repeats. Source data are provided as a Source Data file.

To obtain a high-resolution structure of human Pol III, we isolated the endogenous complex from HeLa cells. To this end, we employed CRISPR/Cas9 genome editing in human cells to create a homozygous knock-in of a cleavable green fluorescent protein (GFP)-tag at the C terminus of subunit RPAC1 (shared between Pol I and Pol III) (Fig. 1a). Fractionation experiments followed by immunopurification using an anti-GFP nanobody revealed that Pol III is present in both nuclear and cytoplasmic fractions (Fig. 1b), in agreement with previous reports highlighting a Pol III cytosolic DNA-sensing activity^17,18^. An optimized large-scale purification, including an ion-exchange step to separate Pol I and Pol III, enabled the isolation of active human Pol III (Fig. 1c, d and Supplementary Fig. 1) from total cell extracts with yields and quality amenable for further EM studies.

### Cryo-EM structure of human Pol III

**Fig. 2:**
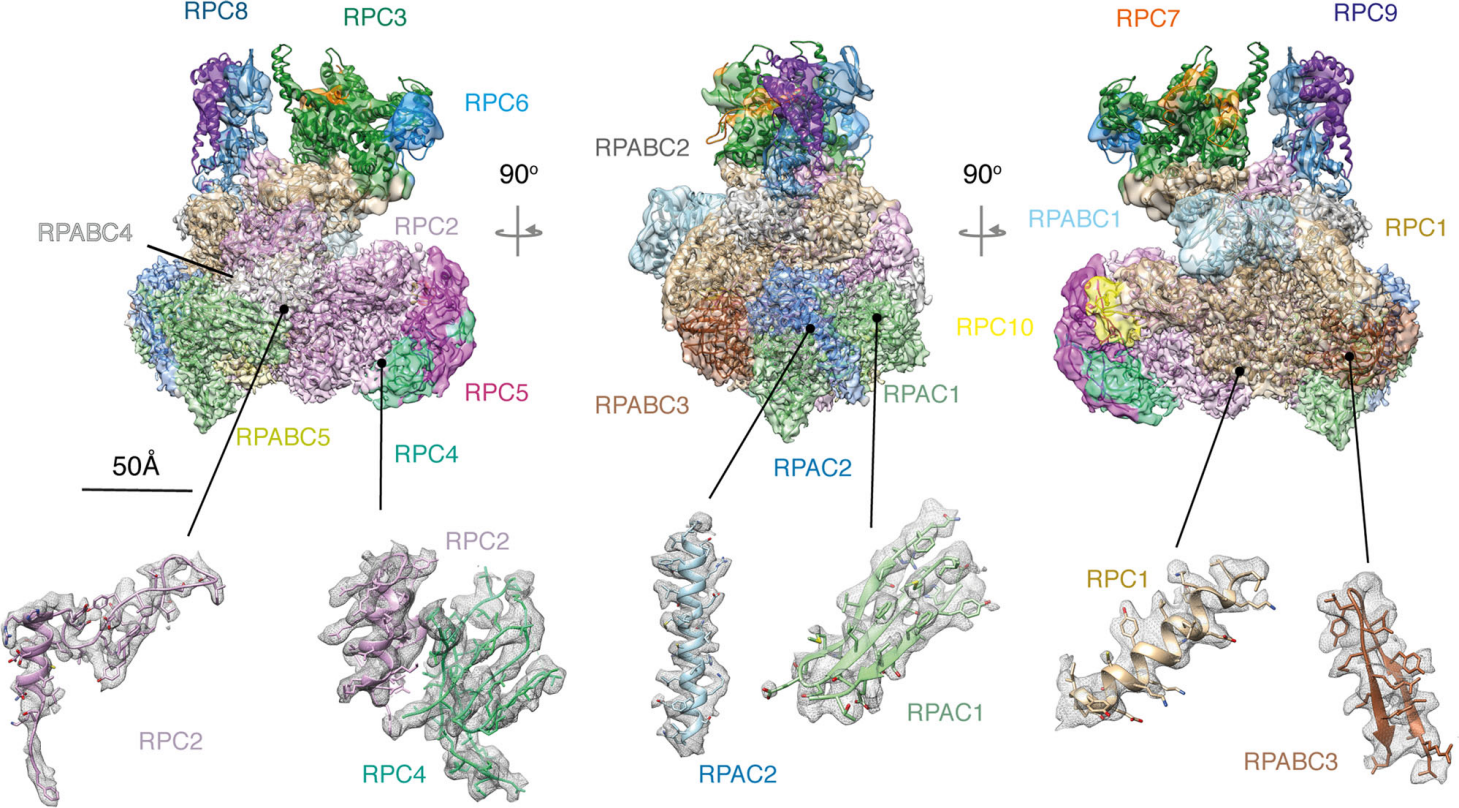
The structure of human RNA polymerase III. Shown is the electron density map filtered according to the local resolution with the fitted model shown in ribbon representation (above). Regions of the electron density map are coloured according to the subunit structure as labelled. Shown below are selected regions of several subunits showing the fit with the filtered electron density (mesh).

The non-crosslinked purified human Pol III sample was applied to carbon-coated cryo-EM grids and imaged on a Titan Krios TEM microscope equipped with a Falcon III camera. Two data sets were collected at 0° and 30° tilting angles, to overcome preferred orientation of the sample on the cryo-grids, resulting in a merged dataset of 172,678 particles after two-dimensional (2D) class averaging (Supplementary Fig. 2 and Table 1). The majority of imaged particles represented the intact 17-subunit human Pol III but a sizeable fraction with a similar angular distribution displayed no density for the RPC3/6/7 heterotrimer, which had possibly dissociated during purification, in agreement with earlier reports^19^, or during cryo-EM specimen preparation. Hierarchical three-dimensional (3D) classification led to a reconstruction of the intact human Pol III from 25,369 particles at an overall resolution of 4.0 Å (Supplementary Figs. 2 and 3, and Table 1). The core of the enzyme is characterized by a very detailed EM map where side chains are clearly discernible (Fig. 2). The RPC8/9 stalk and the RPC3/6/7 subcomplex are more flexible than the core; hence, their local resolution is lower compared to the rest of the complex (Supplementary Fig. 3). Interestingly, the coiled-coil region of the clamp subdomain within the largest subunit RPC1, which is in direct contact with the RPC3/6/7 heterotrimer, also displays a high degree of flexibility. This finding suggests that the coiled-coil region of the clamp together with the heterotrimer form a discrete structural and functional unit, which in yeast has been shown to be able to sense melting of the upstream side of the transcription bubble^4^.

**Table 1:** Cryo-EM data collection, refinement and validation statistics.

Data collection (0° Tilt)	
Voltage (kV)	300
Electron exposure (e^−^/Å^2^)	44.1
Defocus range (μm)	−1.0 to −3.0
Pixel size (Å)	1.065
Data collection (30° Tilt)	
Voltage (kV)	300
Electron exposure (e^−^/Å^2^)	37.8, 40.6
Defocus range (μm)	−1.2 to −3.0
Pixel size (Å)	1.065
Reconstruction (RELION)	
Initial particle images (no.)	172,678
Final particle images (no.)	25,369
Map resolution (Å)	4.0
FSC threshold	(0.143-thr)
Map sharpening *B* factor (Å^2^)	−116.839
Model composition	
Non-hydrogen atoms	34,636
Protein residues	4369
Refinement (PHENIX)	
Map CC	0.53
R.m.s. deviations	
Bond lengths (Å)	0.016
Bond angles (°)	1.086
Validation	
MolProbity score	2.74
Clashscore (all-atom)	31.792
Poor rotamers	0.0
Ramachandran plot	
Favoured (%)	78.66
Allowed (%)	20.94
Disallowed (%)	0.39

As can be expected from the high degree of sequence conservation, the overall structure of human Pol III resembles the yeast counterpart. Structure-based alignments and comparison revealed that most subunits share a high degree of similarity and low root-mean-squared deviation values (Supplementary Fig. 4). However, local differences highlight specific features that might be relevant for human-specific regulation and correct assembly of the Pol III enzyme. Three relevant deletions were observed in the human RPC1 sequence in both the jaw and foot domains. The deletion in the jaw domain removed a small unstructured loop (Supplementary Fig. 5), whereas two deletions in the human RPC1 foot domain result in a rearranged, more compact foot structure (Supplementary Fig. 5). Interestingly, similar structural rearrangements have been observed in the mammalian Pol II foot domain, a region that provides a binding interface for auxiliary regulators such as the DSIF and the PAF complex^20,21^.

In human RPC4, a small deletion removed a helix (residues 269–285), which in the yeast RPC4 protrudes back towards the Pol core and contacts RPC2 (Supplementary Fig. 5). Deletion of this region may therefore highlight a weaker association between the human RPC4/5 heterodimer and core when compared to the yeast enzyme. In the RPC5 dimerization module, structural alignment detected the insertion of a small loop in humans in addition to the large C-terminal insertion (RPC5EXT), which together suggest a slightly rearranged heterodimer module in human Pol III (Supplementary Fig. 5). Furthermore, comparison of the yeast and human stalk subunit RPC9 identified two additional deletions in the human structure which remove unstructured loops (not present in the cryo-EM map of the corresponding yeast subunit). This comparative analysis of the Pol peripheral subcomplexes was limited in the human structure due to the flexibility of the RPC5EXT and heterotrimer-clamp module. As a result, both the reported RPC3 iron-sulphur cluster^22^ and the RPC5EXT, elements absent in the *Saccharomyces cerevisiae* Pol III structures^3,4,23^, were not visible in our EM map.

### Structure of the RPC5 C-terminal extension

**Fig. 3:**
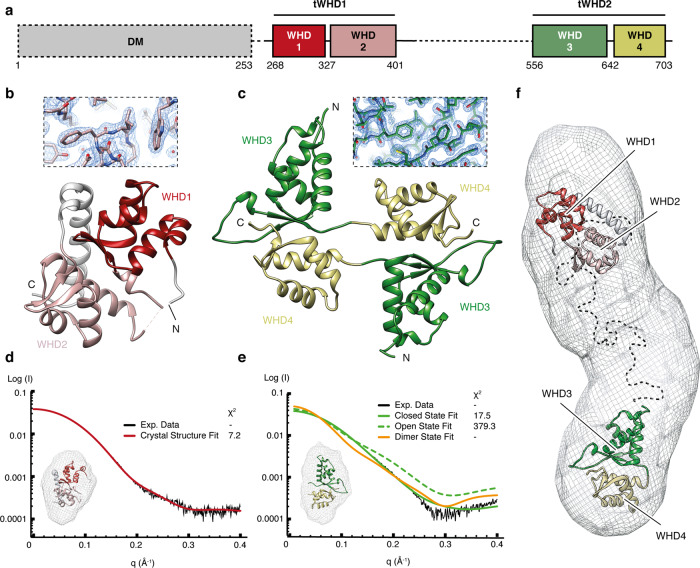
Structure of the RPC5 subunit C-terminal region. **a** Domain architecture of RPC5 C terminus. tWHD1 and tWHD2 are coloured in red and green shades, respectively. Regions are depicted according to their presence (black line) or absence (black dashed line) in the crystal structures. DM refers to RPC5 dimerization module. **b** Crystallographic model of RPC5-WHD1 (dark red), RPC5-WHD2 (light red) and the linker regions (grey) in ribbon cartoon. Inset shows detail of the electron density map. **c** Overall structure of RPC5-tWHD2 crystallographic packing in ribbon cartoon. RPC5-WHD3 and RPC5-WHD4 are shown in dark and light green, respectively, and the inset shows detail of the electron density map. **d** Fitting of RPC5-tWHD1 (red, inset) into the SAXS experimental data (black). **e** Fitting of RPC5-tWHD2 in ‘closed’ (green, inset), ‘open’ (dashed green) and dimer (orange) conformations into the SAXS experimental data (black). **f** Docking of RPC5-tWHD1 (red) and RPC5-tWHD2 (green) into the ab initio SAXS envelope generated from RPC5EXT SAXS data collection.

To gain insight into the structure and function of RPC5EXT, we determined the structure of its individual domains by X-ray crystallography (Supplementary Fig. 6, Fig. 3, and Tables 2 and 3). The RPC5EXT is formed by two consecutive tandem winged helix domains (tWHD1 residues 259–440; tWHD2 residues 556–708) connected by a 115 residue-long flexible linker. Such an architecture has not been reported for other components of the eukaryotic transcription apparatus and appears to be found exclusively in metazoan RPC5. Of the two tWHDs, tWHD1 is the most conserved while tWHD2 is absent in *Caenorhabditis elegans* and *Drosophila melanogaster* (Supplementary Fig. 7). The tWHD1 is formed by two juxtaposed winged helix domains that form a compact globular domain with one of the two recognition helices, typically involved in DNA binding, buried within the structure (Fig. 3b). The compact conformation of tWHD1 is observed also in solution as highlighted by small-angle X-ray scattering (SAXS) data (Fig. 3d and Supplementary Fig. 6). The tWHD2 structure revealed a dimer formed by domain swapping (Fig. 3c). This arrangement is likely caused by the crystallization conditions and, in agreement with this hypothesis, SAXS data showed a monomeric conformation as the most likely in solution (Fig. 3e and Supplementary Fig. 6). Nevertheless, the two possible conformations of tWHD2, compact or elongated, suggests a degree of flexibility within this domain. Finally, SAXS analysis of a construct encompassing the full-length RPC5EXT support the model of two globular compact tWHD domains connected by a long flexible linker, spanning approximately up to 175 Å in length (Fig. 3f and Supplementary Figs. 8 and 9).

**Table 2:** RPC5-tWHD1 (aa. 259–440) data collection, phasing and refinement statistics for MAD (SeMet) structures.

	Crystal 1 (Native)		Crystal 2 (SeMet)	
Data collection				
Space group	P6_1_22	P6_1_22	P6_1_22	P6_1_22
Cell dimensions				
* a*, *b*, *c* (Å)	56.30, 56.30, 275.88	56.43, 56.43, 277.27	56.44, 56.44, 277.17	56.41, 56.41, 276.95
*α*, *β*, *γ* (°)	90, 90, 120	90, 90, 120	90, 90, 120	90, 90, 120
		Peak	Inflection	Remote
Wavelength	0.91983	0.97965	0.97980	0.971970
Resolution (Å)	48.01–2.23 (2.29–2.23)	48.87–2.72 (2.79–2.72)	48.88–2.63 (2.70–2.63)	48.11–2.50 (2.56–2.50)
* R*_sym_ or *R*_merge_	0.05 (0.749)	0.324 (2.163)	0.268 (1.820)	0.246 (1.656)
* I*/σ*I*	26.9 (5.7)	8.7 (1.5)	9.4 (1.5)	8.7 (1.2)
Completeness (%)	100.0 (100.0)	100.0 (100.0)	100.0 (100.0)	100.0 (100.0)
Redundancy	27.4 (28.8)	21.6 (19.6)	21.2 (15.8)	20.0 (10.6)
Refinement				
Resolution (Å)	45.97–2.23 (2.31–2.23)			
No. reflections	376,023			
* R*_work_/*R*_free_	0.1800/0.2193			
No. of atoms	1496			
Protein	1371			
Ligand/ion	4			
Water	121			
*B*-factors				
Protein	50.52			
Ligand/ion	89.50			
Water	54.85			
R.m.s deviations				
Bond lengths (Å)	0.008			
Bond angles (°)	0.84

**Table 3:** Rpc5-tWHD2 (aa. 556–708) data collection, phasing and refinement statistics for MAD (SeMet) structures.

	Crystal 1 (SeMet)		Crystal 2 (SeMet)	
Data collection				
Space group	P1 2_1_ 1	P1 2_1_ 1	P1 2_1_ 1	P1 2_1_ 1
Cell dimensions				
* a*, *b*, *c* (Å)	41.11, 75.76, 62.4	41.37, 75.67, 62.77	41.35, 75.78, 62.79	41.30, 75.72, 62.67
*α*, *β*, *γ* (°)	90.0, 103.57, 90.0	90.0, 103.73, 90.0	90.0, 103.72, 90.0	90.0, 103.64, 90.0
		Peak	Inflection	Remote
Wavelength	0.97942	0.97942	0.97957	0.97174
Resolution (Å)	30.33–1.48 (1.52–1.48)	47.48–1.74 (1.79–1.74)	47.52–1.71 (1.75–1.71)	60.90–1.78 (1.83–1.78)
* R*_sym_ or *R*_merge_	0.075 (0.735)	0.095 (0.942)	0.088 (0.954)	0.118 (1.001)
* I*/σ*I*	11.8 (1.3)	10.5 (1.6)	11.2 (1.5)	9.0 (1.5)
Completeness (%)	96.1 (71.2)	99.9 (100.0)	99.9 (100.0)	99.9 (100.0)
Redundancy	6.1 (3.6)	6.7 (5.8)	6.7 (5.4)	6.8 (6.4)
Refinement				
Resolution (Å)	30.33–1.55 (1.60–1.55)			
No. reflections	364,951			
* R*_work_/*R*_free_	0.1786/0.2078			
No. of atoms	3053			
Protein	2527			
Ligand/ion	8			
Water	518			
*B*-factors				
Protein	24.28			
Ligand/ion	20.98			
Water	36.33			
R.m.s deviations				
Bond lengths (Å)	0.011			
Bond angles (°)	1.15			

**Fig. 4:**
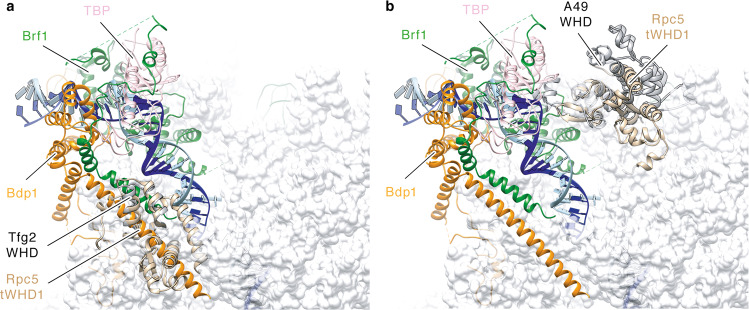
Structural alignment of RPC5-tWHD1 to Pol I and Pol III PICs. **a** Structural alignment between *S. cerevisiae* RNA Pol II (PDB code: 5FYW) and RNA Pol III (PDB code: 6EU0). RPC5-tWHD1 (wheat) superimposition to yeast Tfg2 WHD (grey) leads to clashes with Bdp1 subunit stem motif (orange). **b** Structural alignment between *S. cerevisiae* RNA Pol I (PDB code: 5W66) and RNA Pol III (PDB code: 6EU0). RPC5-tWHD1 (wheat) superimposition to yeast A49 tWHD (grey) does not cause clashes with TFIIIB subunits Brf1 (green), TBP (pink) or Bdp1 (orange).

Comparison with existing protein structures using the Dali server (http://ekhidna.biocenter.helsinki.fi/dali_server/) suggested similarities between tWHD1 and the WHD of *S. cerevisiae* Pol II general transcription factor TFIIF Rap30 subunit^24–26^ and with the tWHD of Pol I A49 subunit^27–31^. Both subunits are orthologs of RPC5 and involved in stabilization of the pre-initiation complexes (PICs), suggesting a putative functional link. However, although the position of TFIIF Rap30 WHD in the Pol II PIC clashes with the Bdp1 subunit of transcription factor TFIIIB in the Pol III PIC^3,4^ (Fig. 4), the equivalent position of A49 tWHD in the Pol I PIC^27^ is accessible in the Pol III PIC (Fig. 4). Thus, one possibility is that, analogously to A49 tWHD, the RPC5-tWHD1 participates in an interaction with the upstream DNA and bound transcription factors, thus stabilizing the human Pol III PIC. In addition, the Dali server analysis retrieved similarities between the individual WHDs of RPC5EXT tWHD2 and the WHDs of cullin and cullin-like proteins, which are involved in ubiquitin-dependent proteolysis^32^.

### The RPC5 extension is required for RNA Pol III stability

**Fig. 5:**
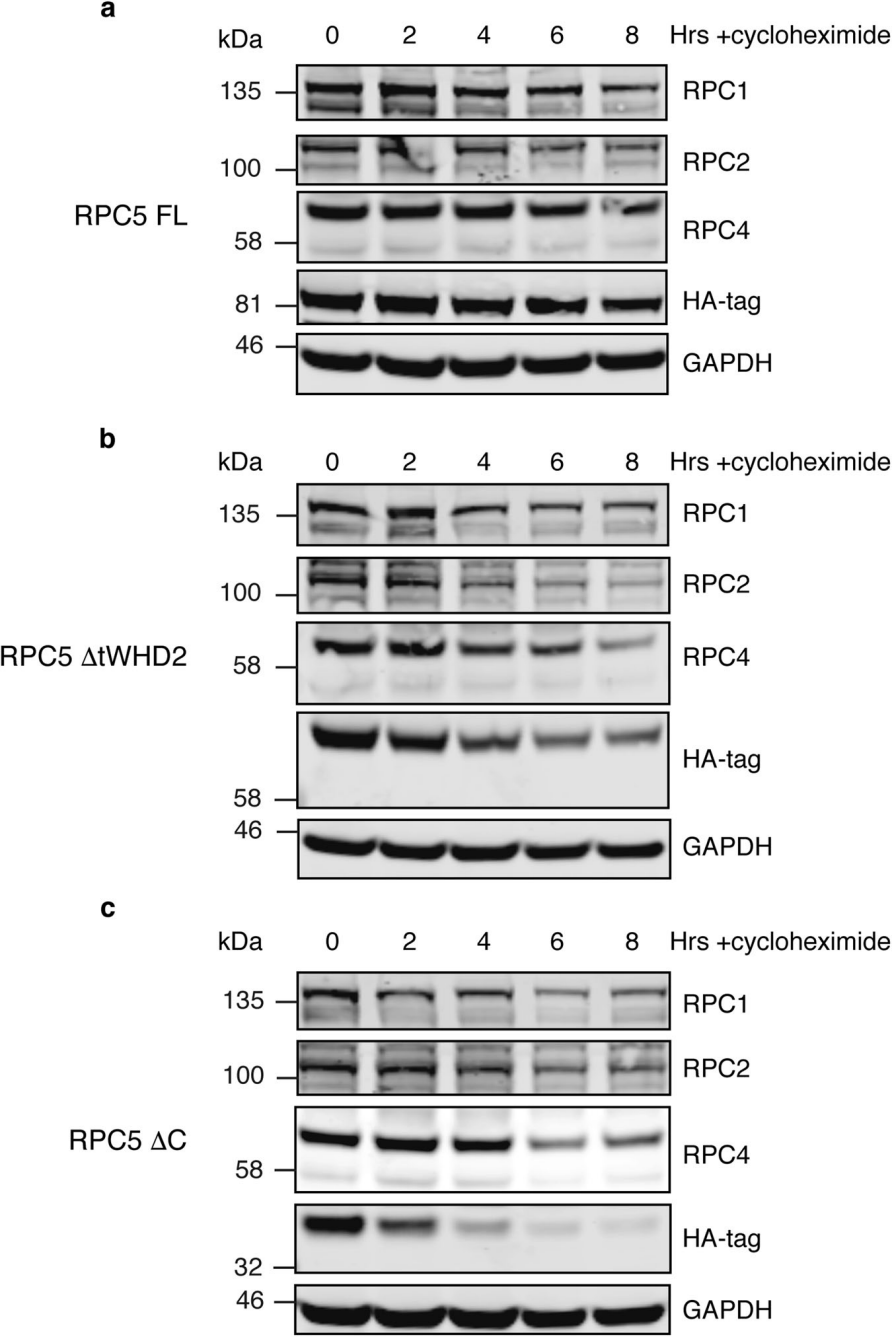
Cycloheximide chase assay investigation of RPC5 protein stability. HEK293T cells were seeded, endogenous RPC5 was knocked down via siRNA and either the FL **a**, ΔtWHD2 **b**, or ΔC **c**, RPC5 constructs were transfected. After 24 h, cycloheximide was added in at a concentration of 300 µg/ml and cells lysed at the specified time points. RPC1, 2, 4 and 5 (as shown via HA-tag antibody) levels were probed by western blotting. Displayed images are representative of three independent experiments. Source data are provided as a Source Data file.

To gain insight into the functional role of RPC5EXT, we used small interfering RNA (siRNA) to knock down RPC5 in HEK293T cells and rescued it with ectopic expression of haemagglutinin (HA)-tagged RPC5 constructs encompassing the full-length protein (RPC5FL) or a version of RPC5 devoid of either tWHD2 (RPC5ΔtWHD2) or the entire RPC5EXT (RPC5ΔC) (Supplementary Fig. 10a, b). Immunoprecipitation using anti-HA magnetic beads revealed that both RPC5FL, RPC5ΔtWHD2 and RPC5ΔC are able to integrate into and pull down a bona fide intact Pol III complex, as probed by RPC1, RPC2 and RPC4 antibodies (Supplementary Fig. 10c). However, the corresponding immunoblots of whole-cell extracts, prior to the immunoprecipitation, indicate lower steady-state levels of RPC5ΔC compared to RPC5FL, pointing towards a direct role of RPC5EXT in enhancing RPC5 stability. To further explore the role of RPC5EXT in regulating RPC5 stability in the context of an intact Pol III complex, we employed a cycloheximide chase assay (Fig. 5). Levels of RPC5FL remained stable for the course of the experiment (8 h), as well as subunits RPC1 and RPC2, suggesting a relatively long half-life of the Pol III complex (Fig. 5a). On the contrary, RPC5ΔtWHD2 and RPC5ΔC were rapidly degraded with RPC5ΔC almost completely depleted after only 2 h following cycloheximide treatment (Fig. 5b, c). Surprisingly, subunits RPC2 and, to a minor extent, RPC1 and RPC4 were also rapidly depleted, suggesting that RPC5EXT is essential for the stability of the whole Pol III complex.

### Pathological genetic mutations map to Pol III subunit interfaces

Many studies have reported mutations of the Pol III enzyme that are related to human diseases, in particular heritable diseases, which affect the correct development of the CNS. Specifically, allele variants encoding mutated versions of the Pol III subunits RPC1, RPC2, RPAC1 and RPAC2 subunits have been established as causative mutations of hypomyelinating leukodystrophy (HL)^7–10,33–37^, Treacher–Collins syndrome (TCS)^11,12^ and Wiedemann–Rautenstrauch syndrome (WRS)^13,14^.

**Fig. 6:**
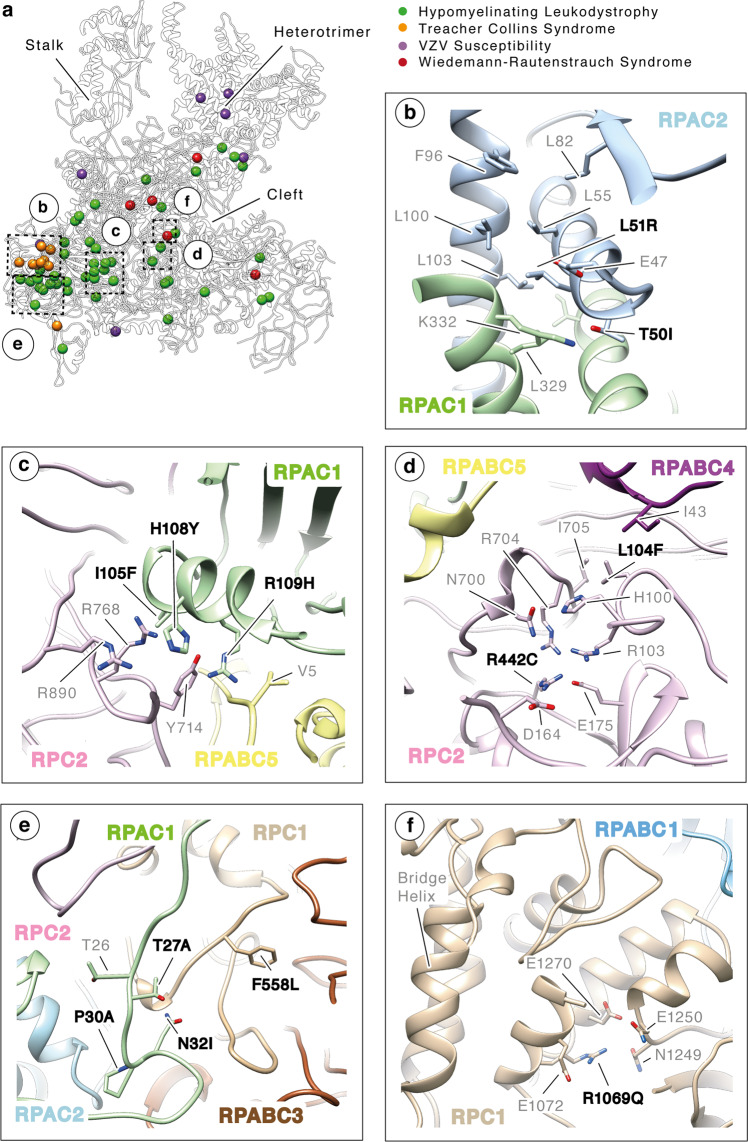
Mapping genetic mutations in human RNA Polymerase III. **a** The position of residues mutated in hypomyelinating leukodystrophy (HL, green), Treacher–Collins syndrome (TCS, orange), Wiedemann–Rautenstrauch syndrome (WRS, red) and VZV susceptibility (VZV, purple) is shown as solid spheres. **b–f** The close-up panels show details of the interfaces involved in TCS (**b**), HL (**c–e**) and WRS mutations (**f**). Side chains are shown in stick representation and Pol III subunits are coloured as in Fig. 2.

To rationalize these findings, we mapped known Pol III mutations on our high-resolution structure (Fig. 6 and Supplementary Data 1). Reported mutations affecting CNS development tend to cluster in specific hotspots, very often at the interface of several Pol III subunits. For example, TCS mutations L51R and T50I in RPAC2 result in disruption of hydrophobic and salt-bridge interactions, respectively, at the interface with the RPAC1 subunit, suggesting a strong destabilizing effect that might impair correct assembly of the enzyme (Fig. 6). Analogously, most reported HL mutations lay at the interface of several subunits and have disruptive effects on these interfaces (Fig. 6 and Supplementary Data 1). Interestingly, WRS mutation R1069Q in subunit RPC1 disrupts a charged interaction with residue N1249 in the same subunit. This residue is itself mutated in HL, possibly altering the interface between subunits RPC1 and RPABC1 in both pathologies. Overall, these finding indicate a general molecular mechanism from mutations resulting in CNS disorders, which is the partial loss of function of Pol III activity through destablization of the enzyme core.

Recently, Pol III mutations have also been described in patients affected by acute severe response to Varicella zoster virus (VZV) infection^15,16,38^. Most of these mutations map at the periphery of the Pol III enzyme and result in neutralizing basic charged residues exposed to the solvent in proximity of DNA-binding regions (Fig. 6). As human Pol III has been shown to display cytosolic DNA-sensing activity, it is conceivable that VZV mutations indeed impair proper DNA binding and transcription factor-independent RNA synthesis in the cytoplasm.

Overall, these findings are in agreement with previous work using the homologous yeast Pol III, and/or Pol I and Pol II enzymes to map disease-causing mutations^8–10,15,39^. However, the availability of a high-resolution structure of human Pol III enable the comprehensive mapping and rationalization of these allele variants with high confidence.

## Discussion

Here we describe the 4.0 Å resolution cryo-EM structure of apo human Pol III. The structure confirms the overall high-degree of structural homology with its *S. cerevisiae* counterpart but also highlights specific differences, such as a rearranged foot domain (Supplementary Fig. 5). Integrating cryo-EM data with X-ray crystallographic and SAXS data for the metazoan-specific RPC5EXT, detailed structural information has been obtained for the whole Pol III complex (Supplementary Fig. 11). Surprisingly, experiments in living cells highlighted a prominent role of RPC5EXT for the integrity of the Pol III complex. Absent in lower eukaryotes, RPC5EXT thus represents an additional metazoan-specific module, impacting on the correct assembly of Pol III. Several abundant phosphorylation sites have been identified in RPC5EXT, which, together with the evidence of structural similarities between RPC5EXT tWHD2 and factors involved in targeted degradation, suggests the intriguing hypothesis of an RPC5EXT-mediated layer of regulation impacting overall Pol III abundance in response to environmental cues that may have evolved in higher organisms.

Furthermore, the high-resolution structure of human Pol III enabled the mapping of more than 85% of reported Pol III genetic mutations with high precision, rationalizing their effects at a molecular level (Supplementary Data 1). Mutations affecting the CNS development tend to spatially cluster together and it seems likely that the severity of the phenotypes observed in HL, TCS and WRS correlate with the disruptive effect of such variants. For example, TCS mutations appear to be particularly disruptive at the interface of RPAC1 and RPAC2, two subunits shared between Pol I and Pol III. Interestingly, mutations N32I and N74S in RPAC1 mutations associated with HL lead to reduced Pol III assembly and nuclear import, without affecting Pol I^7^. This is consistent with our model, as these residues mediate interactions with Pol III-specific RPC1 and RPC2, respectively (Supplementary Data 1).

Finally, as Pol III represents a central nexus involved in the regulation of organismal growth, development and lifespan in eukaryotes and is often deregulated in cancer, the structure of the human enzyme will represent an invaluable tool to aid the design of small molecules capable of specifically targeting Pol III transcription for therapeutic purposes.

## Methods

### CRISPR/Cas9 genome editing

HeLa cells were cultured in Dulbecco’s modified Eagle medium (DMEM, D6429, Sigma Aldrich) supplemented with 10% fetal bovine serum (FBS) (P40-37500, PAN-Biotech) and 1% AAS (A5955, Sigma Aldrich). Genomic integration of Superfolder GFP (sfGFP) ORF at the C terminus of POLR1C was done by CRISPR/Cas9 based on a published protocol (Ran et al.^40^) performed as following.

Design of the guide RNAs (gRNAs) was done with a web-based tool (http:\CRISPR.mit.edu) and annealed oligos (gRNA1 = 5′-gCTAGTTCATCCAAGAAGCGC-3′; gRNA2 = 5′-gCGGTTCAGATGGACTGAGCT-3′) were cloned via BplI into the bicistronic Cas9n expression vector pSpCas9n(BB)−2A-Puro (PX462) V2.0, which was a gift from Feng Zhang (Addgene plasmid #62987: https://n2t.net/addgene:62987; RRID: Addgene_62987). A donor plasmid carried a short GS-linker sequence with an embedded Human Rhinovirus (HRV) 3C protease cleavage site and the sfGFP ORF surrounded by two large sequence segments homologous to the insertion locus in the genome.

HeLa cells were transfected with a 1 : 1 : 1 mix of gRNA1 and gRNA2 vectors together with the donor plasmid using PolyJet transfection reagent (SL100688, SignaGen Laboratories) according to the manufacturer’s instructions. Several days later, the GFP-expressing cells were enriched by flow cytometry using a Bio-Rad S3e cell sorter. GFP-positive cells were seeded on large culture dishes such that they could grow as single cell colonies. After 2–3 weeks, colonies were transferred manually into multi-well slides for live-cell imaging and were screened under identical microscope settings. The brightest clones were selected for expansion. These monoclonal populations were validated by PCR on extracted genomic DNA (using the Blood & Tissue Kit, Qiagen).

The selected cell line was cultivated adherently and adapted to suspension growth as follows: Cells from 8 flasks (about 70 × 10^6^ cells total; 83.3912.302, Sarstedt) were detached by incubation with trypsin (25,300, Gibco) at 37 °C for 5 min, transferred to a spinner flask (250 mL total volume; 4500, Corning) and cultured in suspension with high-glucose DMEM (11965, Gibco) supplemented with 1% FBS (10,270, Gibco) and 1% Penicillin/Streptomycin (P0781, Sigma Aldrich) under moderate stirring at 37 °C, 5% CO_2_ atmosphere. To expand the culture, 1× the current volume of fresh media including all supplements was added when cells reached a density of ~3.5 × 10^5^ cells/mL and transferred to spinner flasks of increasing volume when required. Cells were collected by centrifugation and washed with phosphate-buffered saline (PBS) before flash-freezing the pellet.

For fluorescence imaging, cells were grown adherently on cover slips to 50% confluency. After washing the cells with pre-warmed (37 °C) PBS, they were fixed with 3.7% paraformaldehyde in PBS for 10 min at 37 °C. The fixation was stopped by addition of 100 mM glycine in PBS for 5 min at 37 °C and cells were washed twice with PBS. The cells on the cover slips were mounted on the specimen slide with the help of a drop Prolong Gold Antifade Mountant with 4′,6-diamidino-2-phenylindole (DAPI) (P36941, Thermo Fisher Scientific) and dried for at least 3 days in the dark.

The fluorescent specimens were imaged using a Zeiss Axio Observer.Z1/7 microscope and a ×63 oil-objective lens. DAPI staining was detected with the help of a 405 nm excitation laser and for the emission a wide band-pass filter (300–720 nm) was used. For sfGFP detection, a 488 nm laser and the same band-pass filter (300–720 nm) was applied. The images were captured with the Airyscan mode and detector. Processing was done using the Zeiss AxioVision software, the Zeiss ZEN 3.0 (ZEN lite) software and Fiji.

The selected homozygous cell line was further characterized by western blotting. Cells from a confluent 6 cm plate (about 2.7 × 10^6^ cells, 83.3901.300, Sarstedt) were collected with 300 µl of boiling 1× SDS loading dye (3% (w/v) glycerol, 1.68% (v/v) β-mercaptoethanol, 0.03% (w/v) bromophenol blue, 26 mM Tris pH 6.8, 0.42 % (w/v) SDS) and rigorously shaken at 95 °C for 15 min. Prestained Marker (7719 S, NEB) and 10 µl of sample from the parental and the newly generated cell line were loaded on a SDS gel (NP0223BOX, Thermo Fisher Scientific) and proteins separated by electrophoresis. After blotting (Trans-Turbo Blot, Bio-Rad) on a membrane (1,704,275, Bio-Rad), Ponceau S staining confirmed equal loading. The tagged protein RPAC1 was detected by the primary antibody (sc-374443, Santa Cruz Biotechnology), which was subsequently detected by the fluorescently labelled secondary antibody (926-32210, Li-COR). Prestained Marker and secondary antibody were detected at different wavelengths (Odyssey Infrared Imager Model 9120, Li-COR).

### Anti-GFP pulldown from nuclear and cytosolic fractions

A total of ~7 L POLR1C-GFP Hela cells were grown in a spinner flask (Corning) and collected by centrifugation, yielding a cell pellet of 8.1 g (estimated total of 2.3 × 10^9^ cells). Nuclear extract production was based on a published protocol^41^. The cell pellet was resuspended in 9.8 ml of MC Buffer (10 mM HEPES-KOH pH 7.6, 10 mM KOAc, 0.5 mM Mg(OAc)_2_, 5 mM dithiothreitol (DTT), 0.5 mM phenylmethylsulfonyl fluoride (PMSF)) and incubated for 5 min on ice. Following dounce homogenization, nuclei and cytosolic fractions were separated by centrifugation using a Sorvall SS34 rotor at 18,000 × *g* at 4 °C for 5 min. This resulted in a nuclear pellet of 5.1 g, which was resuspended with 6.7 ml ‘Roeder C Buffer’ (25% v/v glycerol, 20 mM HEPES-KPOH pH 7.9, 1.5 mM MgCl_2_, 0.2 mM EDTA pH 8.0, 420 mM NaCl, 0.5 mM DTT, 0.5 mM PMSF) and corresponds to 12 ml of nuclear extract. Nuclei were lysed with a dounce homogenizer, slowly stirred at 4 °C and centrifuged in a Sorvall SS34 rotor at 16,000 × *g* at 4 °C for 30 min. Nuclear extracts and cytosolic fraction were split into fractions of 0.5 mL each.

An aliquot of nuclear extract and cytosolic fraction were each supplemented with 500 µL wash buffer (5% v/v glycerol, 20 mM HEPES 7.8, 420 mM NaCl, 1 mM MgCl_2_, 0.1 mM ZnCl, 2 mM β-mercaptoethanol, 0.5 mM PMSF) and 5 units DNase I (Thermo Fished Scientific) followed by 30 min incubation at 4 °C in an overhead mixer. Debris was removed by centrifugation (Eppendorf Centrifuge 5427 R, Rotor FA-45-12-17) 13k r.p.m., 4 °C, 30 min. Nuclear extracts and cytosolic fraction were each bound to 20 µL (slurry) ‘GFP selector’ Beads (NanoTag) for 2 h and washed twice with 0.5 mL wash buffer times and washed/eluted as described above. Elution was carried out with 3C protease in 50 µL wash buffer at 4 °C for 2 h. Pre-cast SDS-polyacrylamide gel electrophoresis (PAGE) gels (4–12% NuPAGE Bis-Tris, Thermo Scientific) were loaded with 12.5 µL fractions.

### Large-scale human RNA Pol purification

Large-scale cell growth was carried out at the Cell Services Scientific Technical Platform at The Francis Crick Institute, London. Adherent HeLa POLR1C-GFP cells were grown in DMEM-4 medium supplemented with 1% fetal bovine serum (FCS), 1% Glutamax and 1% Penicillin/Streptomycin. Confluent cells were collected by trypsin treatment followed by gentle centrifugation. The cell pellet was subsequently resuspended in RPMI-1640 supplemented with 5% FCS, 1% Glutamax and 1% penicillin/streptomycin, to allow for cell growth in suspension. Cells were expanded in suspension using a small glass spinner flask flushed with CO_2_ at 37 °C. Cells were expanded to a maximum volume of 1.2 L per growth in a 3 L glass spinner flask. Cells were grown to a density of to 1 × 10^6^ cells/ml with viability maintained at >90%. Following growth, cells were collected by gentle centrifugation at room temperature. The resulting cell pellets were washed with PBS and cells pelleted again via centrifugation. The final cell pellets were stored at −80 °C prior to purification.

For large-scale purification of human RNA Pol, whole-cell lysate was produced from a cell pellet derived from 20 L of HeLa cells grown to 1 × 10^6^ cells/ml density. The cell pellet was resuspended in lysis buffer (50 mM Tris-HCl pH 8.0, 250 mM (NH_4_)_2_SO_4_, 20% v/v glycerol, 1 mM MgCl_2_, 10 μM ZnCl_2_, 10 mM β-mercaptoethanol) and two protease-inhibitor tablets (Roche) added. Lysis was performed through repeated passage of the cell suspension through a dounce followed by sonication with the resulting lysate cleared through centrifugation at 28,000 × *g* at 4 °C for 40 min followed by filtration of the soluble fraction through gauze. The cleared lysate was incubated with 1 ml of GFP selector beads 50% slurry (Nanotag) pre-equilibrated in lysis buffer. Beads were incubated for 3 h at 4 °C under continuous rotation. Beads were washed with 60× slurry volume in lysis buffer and eluted through overnight incubation at 4 °C with 160 μl of HRV−3C protease (Millipore) in a final volume of 2–3 ml. Following elution, the eluate was collected through gentle centrifugation of the beads at 1000 × *g* and collection of the supernatant. The beads were then washed in double the eluate volume with wash buffer (50 mM Tris-HCl pH 8.0, 50 mM (NH_4_)_2_SO_4_, 1 mM MgCl_2_, 10 μM ZnCl_2_, 10 mM β-mercaptoethanol) and the resulting wash fraction combined with the eluate to dilute the (NH_4_)_2_SO_4_ to ~120 mM. The eluate mixture was further diluted through addition of an equivalent volume of Tris buffer (50 mM Tris-HCl, 1 mM MgCl_2_, 10 μM ZnCl_2_, 10 mM β-mercaptoethanol) to reduce the final (NH_4_)_2_SO_4_ concentration to ~60 mM. Next, the eluate was loaded onto a MonoQ GL 5/50 column (GE Healthcare) and eluted in a linear gradient from 60 mM to 1 M (NH_4_)_2_SO_4_ in 50 mM Tris-HCl pH 8.0, 1 mM MgCl_2_, 10 μM ZnCl_2_, 10 mM β-mercaptoethanol. MonoQ purification produced two peaks corresponding to RNA Pol I (eluting at ~380 mM (NH_4_)_2_SO_4_) and RNA Pol III (eluting at ~550 mM (NH_4_)_2_SO_4_). Human RNA Pol III fractions were collected and diluted to a final (NH_4_)_2_SO_4_ concentration of ~110 mM. The sample was then concentrated using a Vivapsin 500 (100,000 Molecular Weight Cut Off to a final concentration of ~0.05–0.1 mg/ml. The concentrated sample was used immediately for grid preparation.

### RNA elongation and cleavage assay

Human Pol III (0.5, 1 or 2 pmol) was preincubated with 0.25 pmol of pre-annealed minimal nucleic acid scaffold (template DNA: 5′-CGAGGTCGAGCGTTGTCCTGGT-3′, non-template DNA: 5′-CGCTCGACCTCG-3′; RNA: 5′-FAM-AACGGAGACCAGGAC-3′) in transcription buffer (20 mM Hepes pH 7.8, 42–168 mM (NH_4_)_2_SO_4_ (hs Pol III buffer), 8 mM MgSO_4_, 10 µM ZnCl_2_, 10% (v/v) glycerol, 10 mM DTT) for 1 h at 20 °C in a 45 µl reaction. For RNA elongation, 10 µmol of each Nucleotide triphosphate was added and the reaction was incubated for 1 h at 28 °C. To examine cleavage activity, the preincubated reaction was incubated for 1 h at 28 °C without the addition of NTPs. In the following, nucleic acid purification was examined by adding 5 M NaCl to an end concentration of 0.5 M and 800 µl 100% ethanol. After precipitation for at least 1 h at −20 °C, the sample was centrifuged for 30 min at 20,000 × *g* and 4 °C. The pellet was washed with 80% ethanol and, after drying, resuspended in 1× RNA loading dye (4 M Urea, 1× Tris-Borate-Ethylenediaminetetraacetic acid, 0.01% bromophenol blue, 0.01% xylene cyanol). The sample was heated to 95 °C for 5 min. As control, 0.25 pmol of scaffold was treated identically, without addition of Pol and NTPs. FAM-labelled RNA product (0.125 pmol) was separated by gel electrophoresis (20% polyacrylamide gel containing 7 M Urea) and visualized with a Typhoon FLA9500 (GE Healthcare).

### Cryo-EM sample preparation and data collection

Human Pol III cryo-EM samples were prepared on C-Flat 1.2/1.3 (400 mesh) grids coated with a thin film of continuous carbon prepared in house. Grids were glow discharged at 15 mA for 30 s using a PELCO EasyGlow instrument prior to sample addition. A 3 μl volume of sample at ~0.06 mg/ml concentration was applied and incubated for 30 s at 18 °C and 100% humidity. Grids were blotted for 1 s at blot force 1 with a 0.5 s drain time and plunge frozen in liquid ethane using the VitroBot Mark IV system (FEI).

Data collection was carried out using a FEI Titan Krios transmission electron microscope (Thermo Fisher) operating at 300 KeV and equipped with a Falcon III direct electron detector (Astbury Biostructure Laboratory, University of Leeds). Separate data collections were carried out for both untilted and 30° tilted data sets. All data sets were imaged using EPU automated acquisition software with the Falcon III operating in electron counting mode at a nominal magnification of ×75,000 and a calibrated sampling of 1.065 Å/pixel. For untilted data collection, 3115 movies were collected. Movies were collected over 45 frames with a 70 s exposure time and a total dose of 44.1 *e*^−^, giving a dose per frame of 0.98 *e*^−^/Å^2^ and a dose rate of 0.63 *e*^−^/Å^2^/s. Data were collected over a defocus range of −1 μm to -3μm. Tilted data collection was carried out at 30^o^ in two separate sessions. The first session collected 921 movies, with a total dose of 37.8 *e*^−^ fractionated over 38 frames during a 70 s exposure, yielding a dose per frame of 0.99 *e*^−^/Å^2^ and a dose rate of 0.54 *e*^−^/Å^2^/s. The second session collected 1703 movies, imaged with a total dose of 40.6 *e*^−^ fractionated over 38 frames during a 70 s exposure. This gave dose per frame of 1.07 *e*^−^/Å^2^ and a dose rate of 0.58 *e*^−^/Å^2^/s. In both tilted data collections, micrographs were collected using a −1.2 to −3 μm defocus range.

### Cryo-EM image processing

Frame alignment and dose weighting was carried out on-the-fly using MotionCor2^42^. Following motion correction, CTFFIND4 implemented in the cisTEM software package was used for contrast transfer function (CTF) estimation^43^. Particle picking was carried out using the ab initio particle picking option in cisTEM^44^ and resulting particles exported to Relion 3.1^45^. Subsequent 2D and 3D classification, refinement and post-processing steps were carried out using Relion 3.1, and ab initio model generation using Cryosparc v2^46^. For the untilted dataset, 332,238 particles were selected, yielding a final particle set of 139,891 particles corresponding to hPol III following multiple rounds of 2D classification. This particle subset was used to generate an initial model of the hPol III structure using the ab initio model functionality in Cryosparc v2. Similarly, 87,075 particles were selected from 2624 30° tilted micrographs, yielding 32,787 particles following 2D classification. Both particle sets were combined generating the merged particle set of 172,678 particles. This was subject to 3D classification in Relion 3.1 using the Cryosparc ab inito model as a reference. Classification produced 5 classes, with a single class (class 4, containing 68,291 particles) corresponding to the complete Pol molecule. This class was refined and subject to CTF refinement. This was a sequential procedure, first correcting for trefoil and fourth-order aberrations, followed by correction for magnification anisotropy in the second step. In the final step, the defocus was refined on a per particle basis to correct for errors in CTF estimation for tilted particles. Following this, a further refinement was carried out, yielding a model at 3.7 Å resolution at the gold-standard 0.143 Fourier shell correlation (FSC) cut-off criterion. Following refinement and post processing, the map was filtered according to the local resolution of each region using the local resolution functionality implemented in Relion 3.1. Inspection of the resulting density revealed poor density and low resolution of the heterotrimer region. To improve this region, a local mask was generated and 3D classification carried out without alignment localized to the heterotrimer. This produced 3 classes, of which 1 (containing 25,369 particles) produced a model with improved heterotrimer density following consensus refinement. This was subject to further CTF refinement, consensus refinement and local resolution estimation to produce the final model reporting 4.0 Å global resolution at the 0.143 FSC cut-off criterion.

### Cryo-EM model building and refinement

As an initial step, homology models were generated for all core (RPC1, RPC2, RPC10, RPAC1, RPAC2, RPABC1, RPABC2, RPABC3, RPABC4 and RPABC5), heterodimer (RPC4 and RPC5) and stalk (RPC8 and RPC9) subunits using the PHYRE2 webserver^47^. These were rigidly fitted into the locally filtered map using the fitted yeast apo-RNA Pol III structure (RCSB Protein Data Bank (PDB) code: 6EU2) as a guide for the relative positioning of the subunits in UCSF Chimera^48^. The placed homology models were then fitted manually to the density using the COOT software package^49^, at this stage regions not present in the EM density were removed from the model. Following manual fitting, the model was fitted using the real-space refinement functionality in PHENIX^50^. The lower resolution of the more dynamic heterotrimer region did not permit the use of this strategy for these subunits. Therefore, the existing crystal structure of human RPC3 in complex with a fragment of RPC7^51^ (RCSB PDB Code: 5AFQ) was structurally aligned with the yeast heterotrimer in the yeast apo-RNA Pol III structure in UCSF Chimera. Comparison revealed a highly similar structure and relative arrangement for the RPC3 and RPC7 regions present in both structures. Further to this, a homology model for RPC6 was generated using PHYRE2. This was structurally aligned in UCSF Chimera to the yeast RPC6 subunit present in the yeast apo Pol III structure. The region of human RPC6 consisting of residues 174–289 was selected for inclusion in the model, as this was the region which corresponded to residues 171–271 of yeast RPC6, which were visible in the yeast RNA Pol III apo state^4^. Following selection of the relevant models, they were positioned using the yeast apo structure as a guide and then fitted to the human EM map using the sequential fit option in UCSF chimera.

### RPC5 cloning, expression and purification

Based on secondary structure predictions using PsiPred^52^ and HHPred programmes^53^, we designed 13 different constructs of the C-terminal extension of RPC5 subunit (Uniprot ID Q9NVU0). A PCR-based strategy was used to amplify fragments of RPC5-tWHD1, RPC5-tWHD2 and RPC5EXT from its genomic DNA (Genscript). The constructs were subsequently cloned into pOPINF or pOPINJ plasmids for bacterial expression or into pACEBac1 plasmid for baculovirus-insect cells expression. Two hexahistidine-tagged constructs that rendered high yield expression of undegraded proteins (RPC5 (259–440) and RPC5 (556–708)) were selected for large-scale production. Both protein constructs were expressed and purified following the same protocol. Cells were grown at 37 °C, 200 r.p.m. in Terrific Broth to OD_600_ = 1.5 and protein expression was induced with 1 mM isopropyl β-d-1-thiogalactopyranoside at 20 °C overnight. All subsequent steps were performed at 4 °C. Collected cells were resuspended in 20 mM HEPES pH 7.9, 150 mM NaCl, 10 mM imidazole and 10 mM β-mercaptoethanol supplemented with DNAse I and two protease-inhibitor tablets (Roche). After a 30 min incubation, the sample was sonicated and fractionated by centrifugation at 20,000 r.p.m. for 40 min. Then, the soluble fraction was loaded in a HisTrap HP 5 mL affinity column (GE Healthcare) pre-equilibrated with lysis buffer. After extensive washes of the chromatographic column, the protein was eluted with lysis buffer supplemented with 250 mM imidazole. The sample was diluted to 70 mM NaCl and injected into an HiTrap Heparin HP 5 ml (GE Healthcare) column equilibrated with 20 mM HEPES pH 7.9, 70 mM NaCl and 10 mM β-mercaptoethanol. The protein was subsequently eluted using an isocratic gradient from 70 mM to 2 M NaCl in 30 column volumes. Fractions containing RPC5 constructs were identified by SDS-PAGE analysis. Cleavage of the His-tag was performed overnight incubating the protein with 3C protease in a 1 : 50 molar ratio (3C protease: RPC5). Uncleaved His-tagged proteins were removed by incubation of the sample with 1 ml HisPur^TM^ (Thermo Fisher) nickel resin for 1 h at 4 °C. The cleaved protein was concentrated to 5 ml and loaded in a HiLoad 16/600 Superdex 75 pg gel-filtration column (GE Healthcare) equilibrated with 50 mM Tris-HCl pH 7.5, 150 mM NaCl and 10 mM β-mercaptoethanol. Purified RPC5 (259–440) and RPC5 (556–708) were concentrated to 30 and 80 mg/ml, respectively, flash-frozen and stored at −80 °C.

The expression of selenomethionine-derivatized proteins was performed in the methionine-auxotroph *Escherichia coli* B834(DE3) strain (Novagen) using SelenoMet^TM^ medium (Molecular Dimensions) supplemented with SelenoMet Nutrient Mix and 40 mg/l l-selenomethionine (SeMet). Purification of the proteins was performed as described for the native proteins.

The expression of the whole C-terminal extension of RPC5 (referred as RPC5EXT) was performed in the insect cells/baculovirus expression system. Large-scale suspension cultures (300 mL) of High Five insect cells at 0.5 × 10^6^ cells/ml were grown in Insect-Xpress media (Lonza) and inoculated with P2 baculovirus solution containing the RPC5 constructs. Proliferation arrest was assessed by measurement of GFP production until fluorescence reached a plateau. Cells were collected at 800 × *g* for 5 min and the pellets were stored at −20 °C. After a milder sonication step, purification was performed following the protocol described above. Finally, protein was concentrated to 10 mg/mL, flash-frozen in liquid nitrogen and stored at −80 °C.

### Crystallization, data collection and structure determination

Crystals used for structure determination were grown from a 1 : 1 ratio solution (protein : reservoir) using the vapour diffusion technique at 4 °C. RPC5 (259–440) crystals in P6_1_22 space group were obtained at 30 mg/mL after 3–4 days equilibration in 3.2 M NaCl, 100 mM Sodium Acetate pH 4.6 and 10 mM ZnCl_2_. SeMet-RPC5 (259–440) crystals in the same space group were obtained at similar conditions but required the use of streak seeding with diluted native crystals to favour nucleation. RPC5 (556–708) crystals in P2_1_ space group grew at 35 mg/mL in 3.2–3.8 M Ammonium Acetate and 100 mM Bis-Tris Propane pH 6.5–7 after 2 weeks. Crystallization of SeMet-RPC5 (556–708) protein was performed under identical conditions. All crystals were flash-frozen in liquid nitrogen using perfluoropolyether oil (Hampton Research) as a cryoprotectant.

A dataset from native RPC5 (259–440) was collected at 0.9198 Å wavelength in I24 beamline of Diamond Light Source (DLS). In addition, multi-wavelength anomalous dispersion (MAD) data collections were performed at the peak, remote and inflection wavelengths from SeMet-derivatized crystals of RPC5 (259–440) and RPC5 (556–708) in I03 beamline of DLS. Using the MAD dataset, an initial model of SeMet-RPC5 (259–440) at 2.7 Å was determined with the SHELXC/D/E suite from the HKL2Map programme (for phase determination) and the Buccaneer software (for model building). The native structure at 2.2 Å was solved by molecular replacement using the initial SeMet model as a search reference in PHENIX.automr. Subsequent refinement was performed using COOT and PHENIX suites. The structure of RPC5 (556–708) at 1.48 Å was solved from the SeMet data sets using HKL2Map and Buccaneer programmes, and further refined to acceptable *R*_free_/*R*_work_ values with COOT and PHENIX. Protein secondary structure assignment from the atomic coordinates was performed using STRIDE^54^.

### RPC5 SAXS data collection and processing

SAXS data collection was carried out at the SWING small- and wide-angle scattering beamline, SOLEIL Synchrotron, Saint Aubin, France. Purified RPC5EXT (164 μM), tWHD1 (922 μM) and tWHD2 (3691 μM) were passed through a Bio SEC-3 HPLC column (Agilent) at 0.2 ml/min in 50 mM Tris-HCl pH 7.5, 150 mM NaCl, 10 mM β-mercaptoethanol with protein elution monitored using A_280_. Data were collected using an Eiger X 4 M detector (Dectris) at a 2 m distance, using a *q*-range of 0 < *q* < 0.68 Å^−1^. Data were reduced and buffer subtraction performed at the beamline. Data analysis was carried out using the ScÅtter software package for determination of radius of gyration (*R*_g_), *P*(*r*) distribution, particle maximum dimension (*D*_max_) parameters and for qualitative flexibility analysis (through generation of *R*_g_-Normalized Kratky, SIBLYS and Porod-Debeye plots)^55^. Volumetric bead modelling was performed using the DAMMIN software package^56^. Briefly, ab inito bead models were calculated using DAMMIN ten times for each construct, fitting over the *q*-range of 0 < *q* < 0.25 Å^−1^. The resulting bead models were averaged and filtered using the DAMAVER package^57^, generating the final bead model reconstruction.

Comparison of the theoretical scatter profiles of the determined crystal structures for the tWHD1 and tWHD2 constructs was performed using the CRYSOL package for structural validation^58^. Modelling of the entire RPC5 C terminus (RPC5EXT) was performed using Ensemble Optimisation (EOM) analysis from the ATSAS package^59^ of the RPC5EXT SAXS data using the *q*-range 0 < *q* < 0.2 Å^−1^. The determined tWHD1 and tWHD2 crystal structures were defined as rigid bodies in the RPC5 C-terminal sequence, with EOM analysis modelling the intervening 115 amino acid linker as dummy atoms, generating a pool of 10,000 random structural conformations from which the ensemble was selected to sample the continuous structural heterogeneity.

### Cell culture

HEK293T cells (a kind gift from Dr Sebastien Guettler) were cultured in DMEM supplemented with 10% heat-inactivated FBS and 1% penicillin/streptomycin at 37 °C in 5% CO_2_. For transient knockdown, 20 nM siRNA of either ONTARGETplus siRNA for RPC5 (Horizon Discovery) or AllStars negative control siRNA (Qiagen) was used per well. This was transfected using Lullaby transfection reagent (Oz Biosciences) as per the manufacturers’ instructions. The sequences for each RPC5 siRNA are as follows: 5′-UGGAUAAGGCUGACGCCAA-3′, 5′-GGGAGCAGAUUGCGCUGAA-3′, 5′-CGACGAGACCAGCACGUAU-3′, 5′-CCUCGAUGACCUACGAUGA-3′. For transient overexpression of RPC5 (HA-tagged full-length ΔtWHD2 or ΔC), DNA (1.5 μg) was transfected in using Fugene HD transfection reagent (Promega) as per the manufacturers’ instructions.

### Antibodies

The following primary antibodies were used: POLR3A (ab96328, Abcam), POLR3B (ab137030, Abcam), POLR3D (ab86786, Abcam), POLR3E (ab134560, Abcam), HA-tag (ab9110, Abcam), GAPDH (MAB374, diluted 1 : 5000 for western blotting, Merck) and RPA40 (sc-374443, Santa Cruz). The following secondary antibodies were used: anti-rabbit IgG (H + L) DyLight^TM^ 800 4× PEG conjugate (#5151, Cell Signalling Technology) and anti-mouse IgG (H + L) DyLight^TM^ 680 (#5470, Cell Signalling Technology). All antibodies were used at a dilution of 1 : 1000, unless otherwise stated.

### Co-immunoprecipitation and western blotting

HEK293T cells were seeded into 10 cm plates in the presence of siRNA. After 24 h, they were subsequently transfected with RPC5 (HA-tagged full-length ΔtWHD2 or ΔC) and maintained for a further 24 h. Cells were lysed in RIPA buffer and co-immunoprecipitation was performed using Pierce^TM^ anti-HA magnetic beads (Thermo Fisher Scientific). HA-tagged proteins were eluted from the beads through addition of NuPAGE^TM^ LDS 4× sample buffer and boiling the samples for 10 min. For whole-cell lysates, cells were lysed in RIPA buffer and then NuPAGE^TM^ LDS 4× sample buffer (Thermo Fisher Scientific) plus NuPAGE^TM^ 10× sample reducing agent (Thermo Fisher Scientific) was added before being boiled for 5 min. SDS-PAGE was subsequently performed on the lysates in 4–12% Bis-Tris protein gels, transferred to nitrocellulose membrane, blocked for 1 h in 5% milk/Tris-buffered saline/0.1% Tween20 and probed with primary antibody overnight at 4 °C. Secondary antibodies were incubated for 1 h at room temperature in the dark and detected using the Odyssey-CLx fluorescence imaging system (LI-COR Biosciences). All uncropped gel images are available in the source data file.

### Cycloheximide chase assay

HEK293T cells were seeded into six-well plates in the presence of siRNA. After 24 h, they were subsequently transfected with either RPC5 (HA-tagged full length or ΔC) and maintained for a further 24 h. Cycloheximide was then added at a concentration of 300 μg/ml and cells lysed at regular time points (every 2 h, up to a maximum of 8 h) using RIPA buffer and lysates were analysed via western blotting, as previously stated. All uncropped gel images are available in the Source Data file.

### Reporting summary

Further information on research design is available in the Nature Research Reporting Summary linked to this article.

## Supplementary information

Supplementary Information

Peer Review File

Description of Additional Supplementary Files

Supplementary Data 1

Reporting Summary

## Data Availability

The electron density reconstructions and final model were deposited with the Electron Microscopy Data Base under accession code number EMD-11904 and with the Protein Data Bank (PDB) under accession code 7AST. The PDB accession numbers for the atomic coordinates and structure factors of the RPC5EXT tWHD1 and tWHD2 crystal structures reported in this paper are 7ASU and 7ASV, respectively. The RNA Pol models used in this study are available from PDB under accession codes 5FYW, 5W66, 6EU0, 6EU2 and 6EU3. Source data are provided with this paper.

## Source data

Source Data
