# Supplementary material for: Structure of human RNA polymerase III
Source: Nat Commun. 2020 Dec 17;11:6409. doi: 10.1038/s41467-020-20262-5 (PMC7747717; doi:10.1038/s41467-020-20262-5)
Supplement: Supplementary file 1 — Supplementary Information [file 41467_2020_20262_MOESM1_ESM.pdf]

Supplementary Information

Structure of human RNA Polymerase III

Ewan Phillip Ramsay[1†], Guillermo Abascal-Palacios[1†], Julia L. Daiß[2†], Helen King[1], Jerome Gouge[1], Michael Pilsl[2], Fabienne Beuron[1], Edward Morris[1], Philip Gunkel[3], Christoph Engel[2*] and Alessandro Vannini[1,4*]

Affiliations:

[1]Division of Structural Biology, The Institute of Cancer Research, London SW7 3RP, United Kingdom.

[2]Regensburg Center for Biochemistry, University of Regensburg, 93053 Regensburg, Germany

[3]Max Planck Institute for Biophysical Chemistry, Research Group Nuclear Architecture, 37077 Göttingen, Germany

[4]Fondazione Human Technopole, Structural Biology Research Centre, 20157 Milan, Italy

[†]These authors contributed equally to this work

*Corresponding authors

*email: christoph.engel@ur.de, alessandro.vannini@fht.org

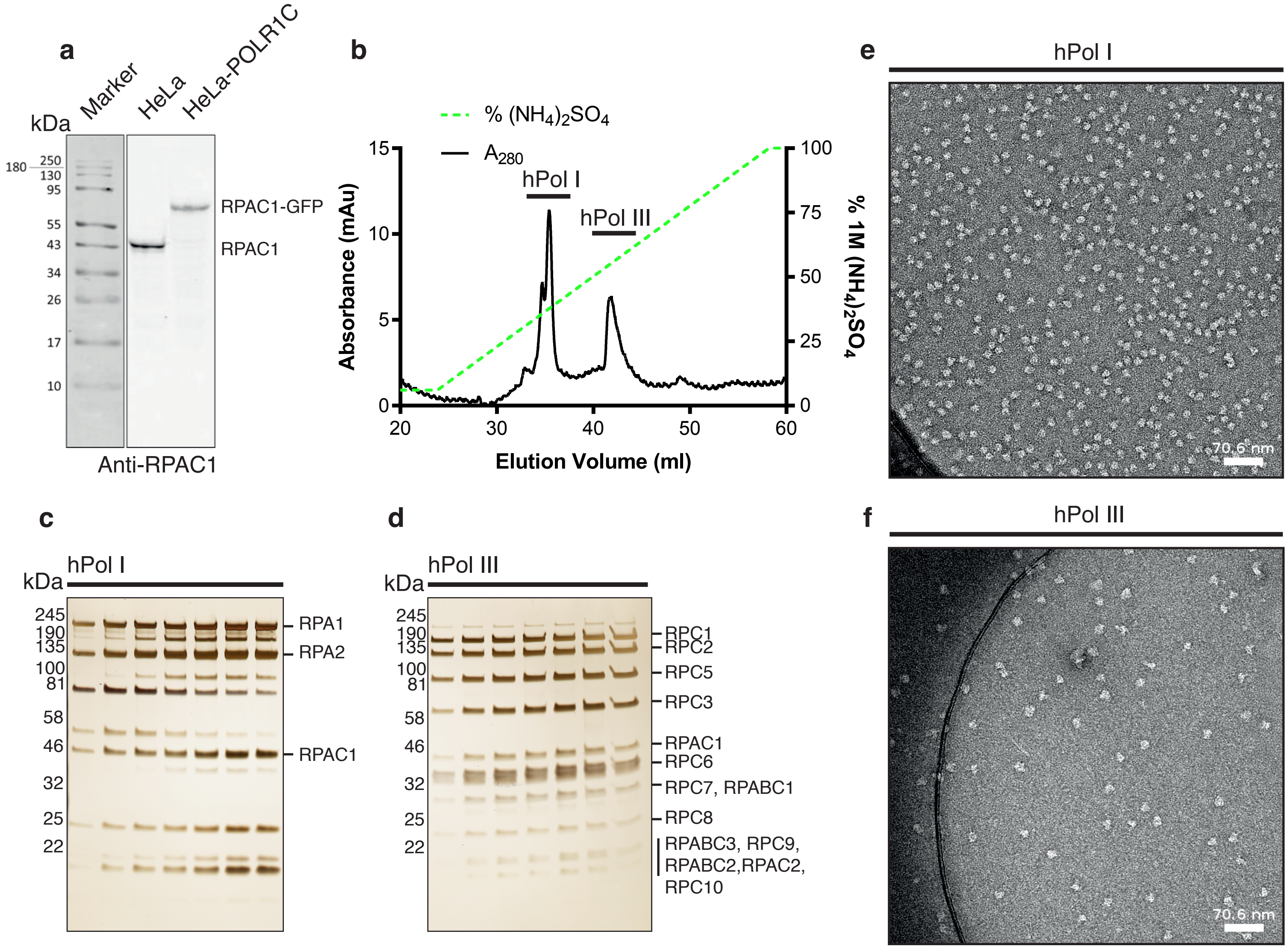

Supplementary Figure 1 - Purification of endogenous human RNA Pol III. (a) Western blot of HeLa and HeLa POLR1C-GFP whole cell lysate probed with anti-RPAC1 antibody. Marked is signal corresponding to the RPAC1 RNA polymerase subunit, insertion of the GFP tag leads to the expected increase in molecular weight. This is a representative image of two individual experiments. (b) MonoQ chromatogram trace of GFP affinity chromatography eluate. Marked are two species eluting at ~380mM $(NH_4)_2SO_4$ and ~550mM $(NH_4)_2SO_4$ corresponding to purified human RNA polymerase I (hPol I) and human RNA polymerase III (hPol III) respectively. (c) Silver stained SDS-PAGE analysis of MonoQ fractions derived from the hPol I and (d) hPol III species, marked are polymerase subunits. Shown are excised regions corresponding to hPol I and hPol III lanes from a representative image of three independent purifications. (e) Representative negatively stained electron micrograph of purified hPol I (from a total of 180 collected micrographs) and (f) hPol III (from a total of 217 collected micrographs). All source data are provided as a Source Data file.

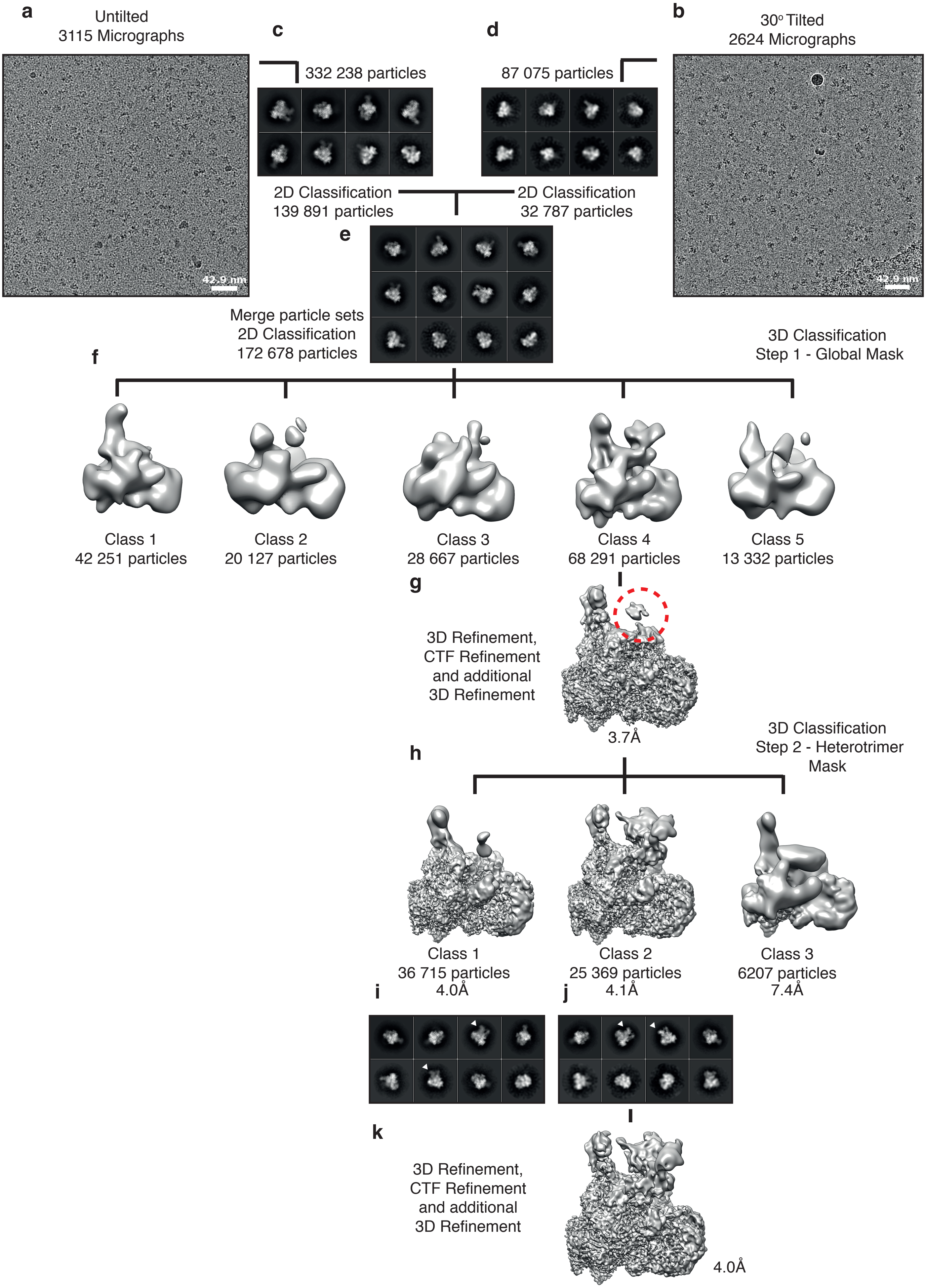

Supplementary Figure 2 - Human Pol III cryo-EM data processing. (a) Representative micrograph (from 3115 micrographs) for untilted and (b) 30° tilted datasets (from 2624 micrographs). Representative 2D class averages for (c) untilted, (d) 30° tilted and (e) merged datasets. (f) Consensus 3D classification of the resulting particle set using a cryosparc ab inito model as reference. The class corresponding to full polymerase was refined (g) and then subject to masked classification around the heterotrimer (marked with red circle) (h). (i,j) Representative 2D classes of resulting classes, with the heterotrimer density marked. The class corresponding to the full polymerase molecule was subsequently refined to generate the final model (k).

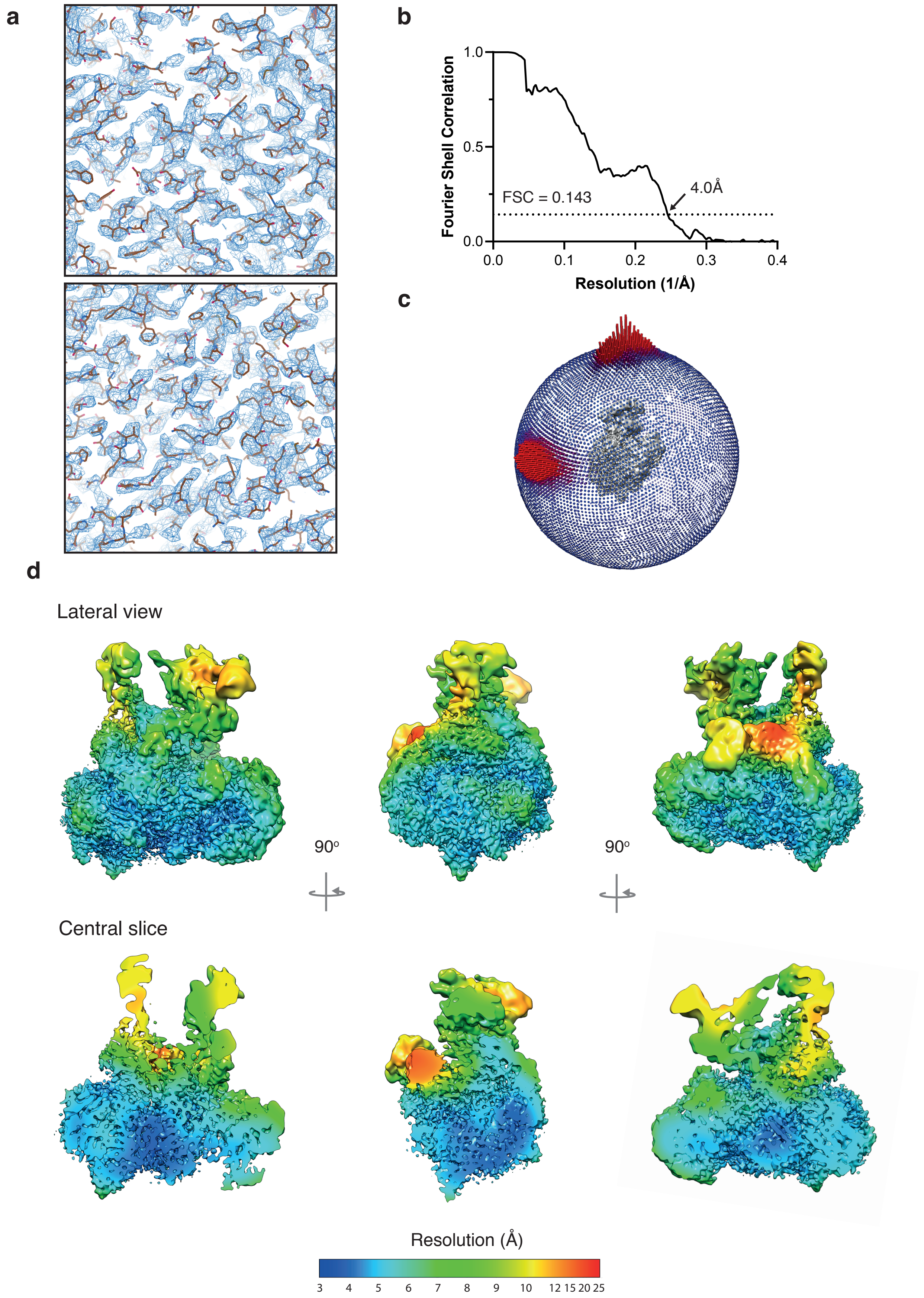

Supplementary Figure 3 - Resolution determination of human Pol III cryo-EM reconstruction. (a) Central slices of the refined map with model fitted. (b) Fourier shell correlation (FSC) of the final human polymerase III reconstruction, reporting a resolution of 4.0Å at 0.143 FSC. (c) Lateral view of the orientation distribution of the particles which contributed to the reconstruction. The heights of the bars reflect the relative number of particles in each orientation. (d) Lateral view (above) and central slice (below) displaying the local resolution distribution of the final refined map. Map density is filtered and coloured according to the local resolution estimation, as indicated by the colour key (below). Local resolution was estimated using the Relion-3.1 package.

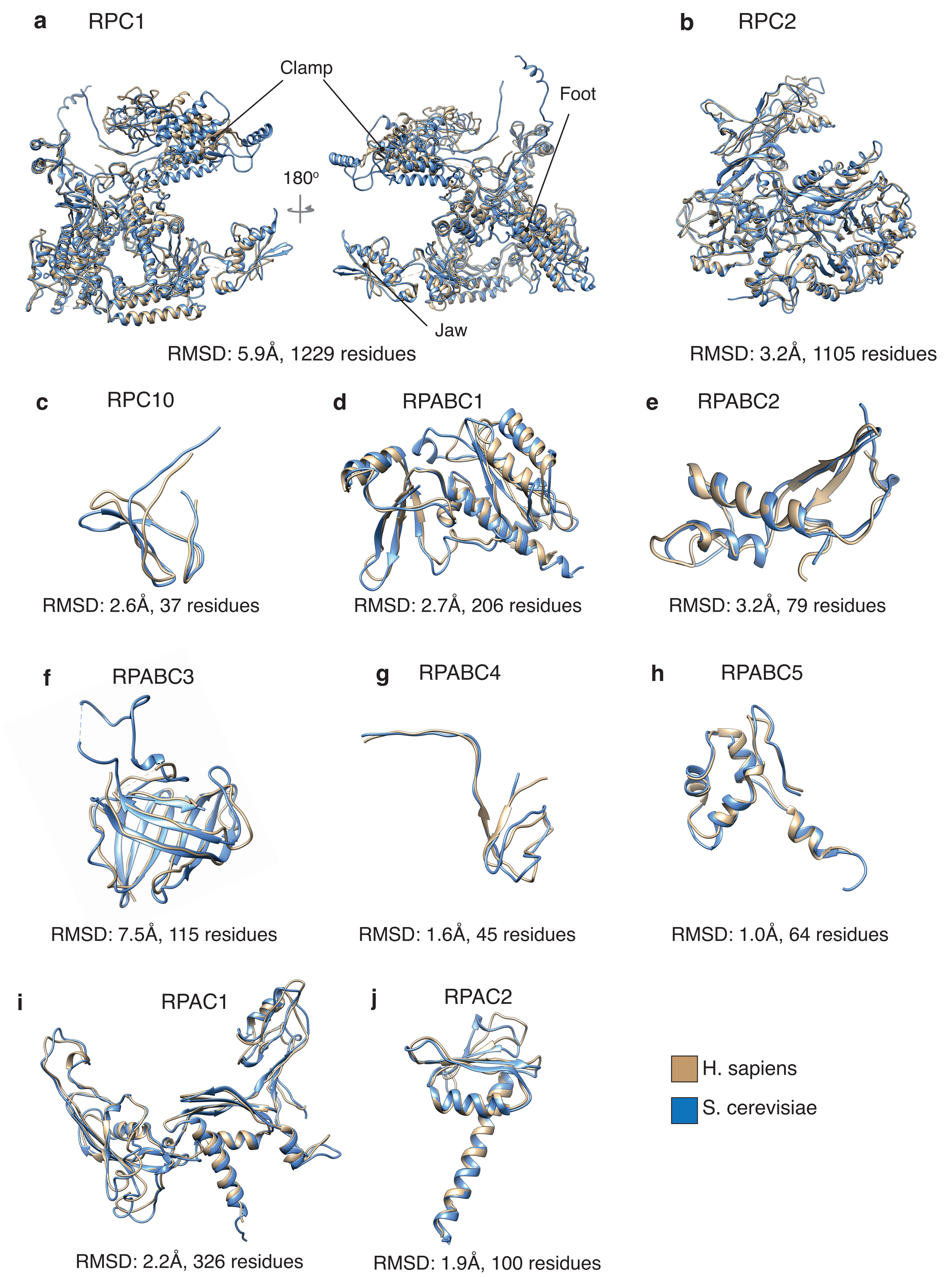

Supplementary Figure 4 - Structural alignment of human and yeast (6EU3) RNA pol III core subunits reveals high structural similarity in the polymerase core. (A-J) Shown are structural alignments between the human (tan) and yeast (blue) polymerase core subunits with the reported RMSD values of the alignment shown below. In each case, structural alignment revealed a highly similar fold for all core subunits.

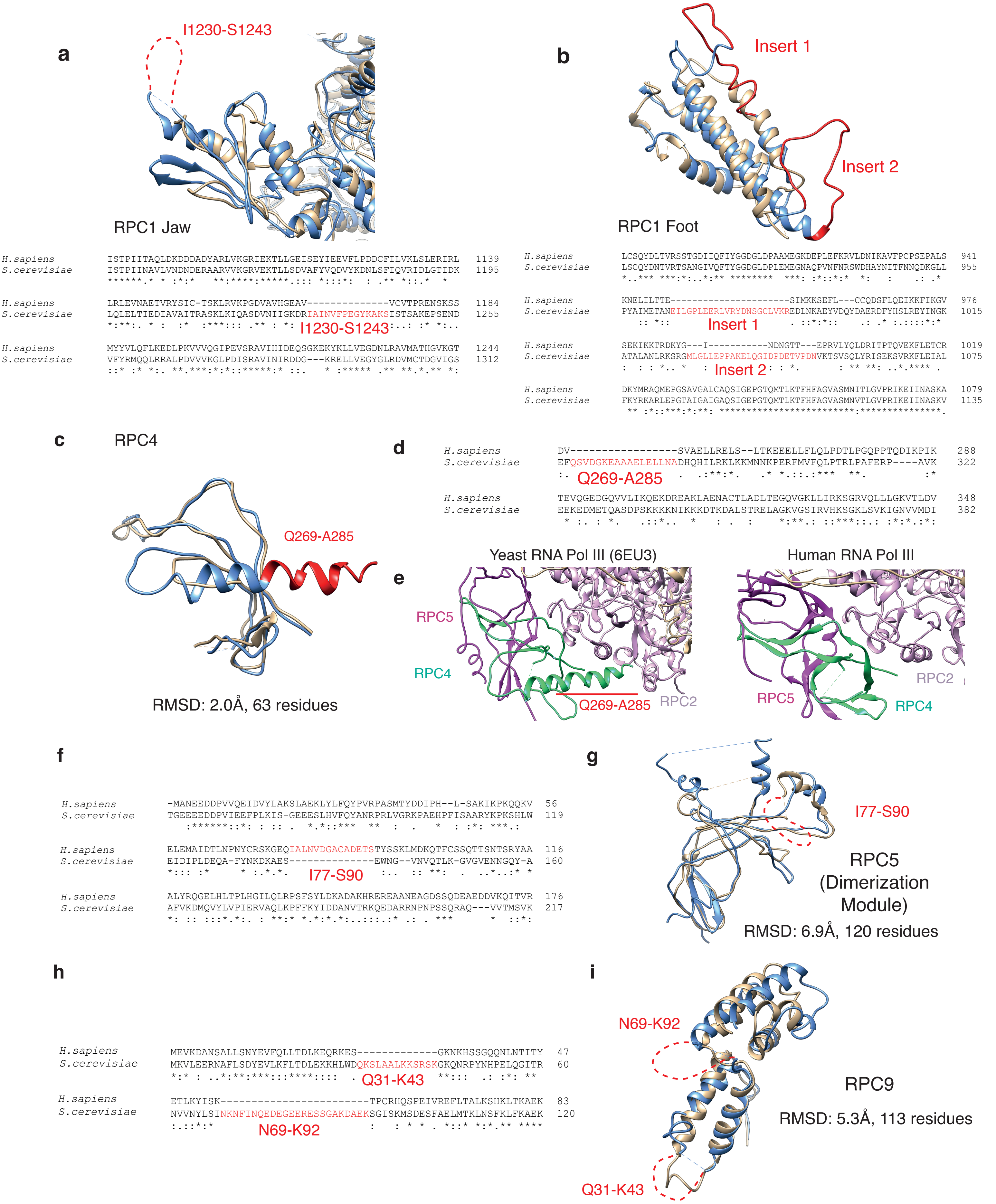

Supplementary Figure 5 - Structural differences identified between human and yeast Pol III. In all comparisons the human structure is rendered in tan with the yeast in blue and the RMSD values reported, insertions are highlighted in red. Shown are structural (top) and sequence (bottom) alignments of the (a) RPC1 jaw and (b) RPC1 foot domain, showing the sites of deletion in the human subunit (in red). (c) Structural and (d) sequence comparison of human and yeast RPC4 shows high similarity apart from a deletion of a small alpha-helical segment (red) in the human subunit, which protrudes back towards the polymerase core in yeast (e, left) but not human (e, right) Pol III. Both sequence (f) and structural (g) aligment of the RPC5 dimerisation module identifies an additional insertion in the human RPC5 subnuit (highlighted in red). Sequence (h) and structural (i) alignment of yeast and human RPC9 detects two deletions of unstructured loops from the human sequence in the polymerase stalk (highlighted in red).

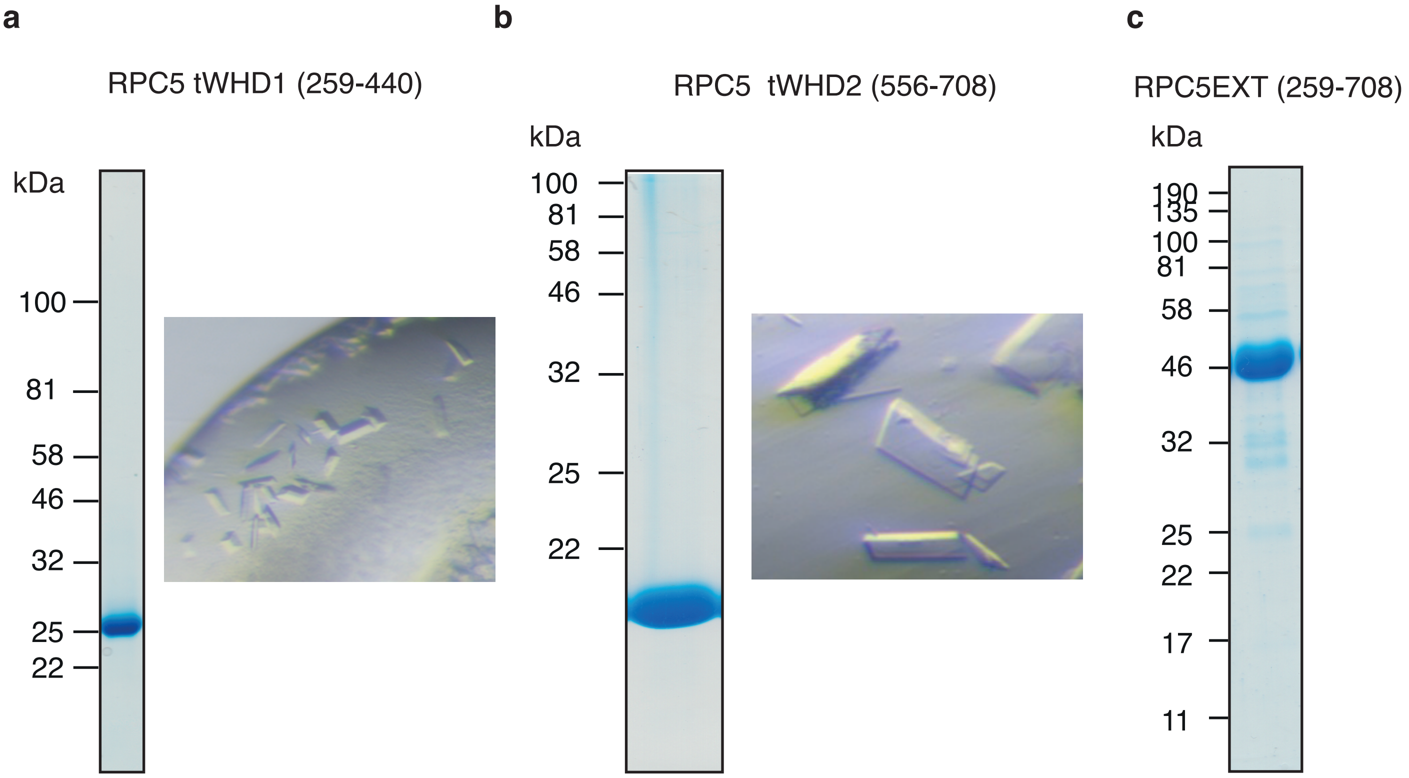

Supplementary Figure 6 - Purification of RPC5 C-terminal constructs. (a) Coomassie stained SDS-PAGE gel (left) and crystals (right) of purified RPC5 tWHD1 construct. (b) Coomassie stained SDS-PAGE gel (left) and crystals (right) of purified RPC5 tWHD2 construct. (c) Coomassie stained SDS-PAGE gel showing purified RPC5EXT construct used for SAXS analysis. Shown are excised lanes froom representative gel images of three independent purifications. All source data are provided as a Source Data file.

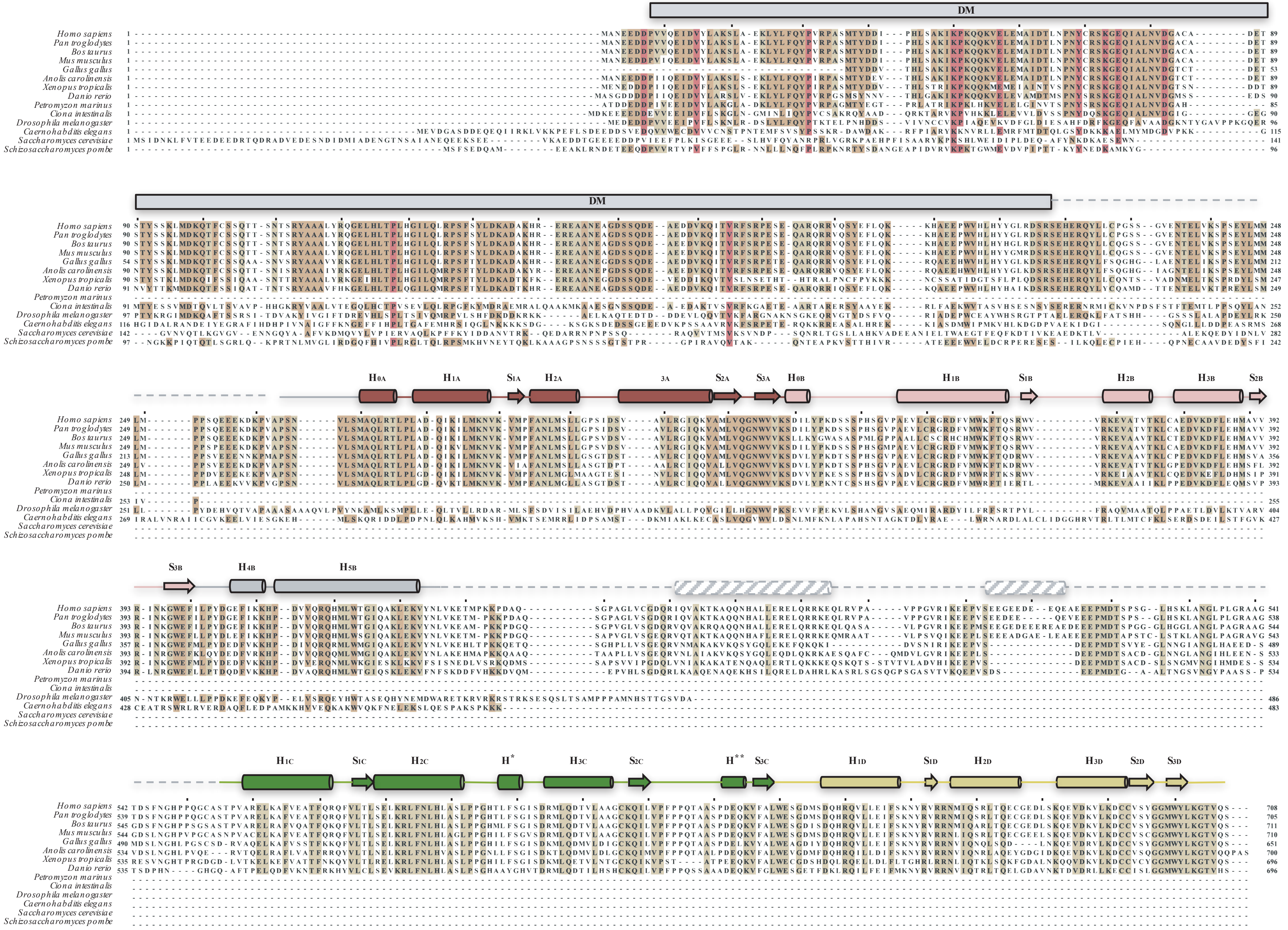

Supplementary Figure 7 - RPC5 sequence and architecture conservation. Multiple sequence alignment (MSA) of human RPC5 with representative organisms. Residues are coloured according to the identity percentage: >80% (red), >60% (dark brown) or >40% (light brown). A schematic representation of the known and predicted structural elements is depicted above the MSA. Solved structures of the linkers (grey), RPC5-tWHD1 (red) and RPC5-tWHD2 (green) are shown as cylinders (α-helixes) or arrows (β-strands). The secondary structure prediction of RPC5 linker (between tWH1 and tWH2) is shown in dashed grey. Rpc5 dimerization module is shown as a grey box.

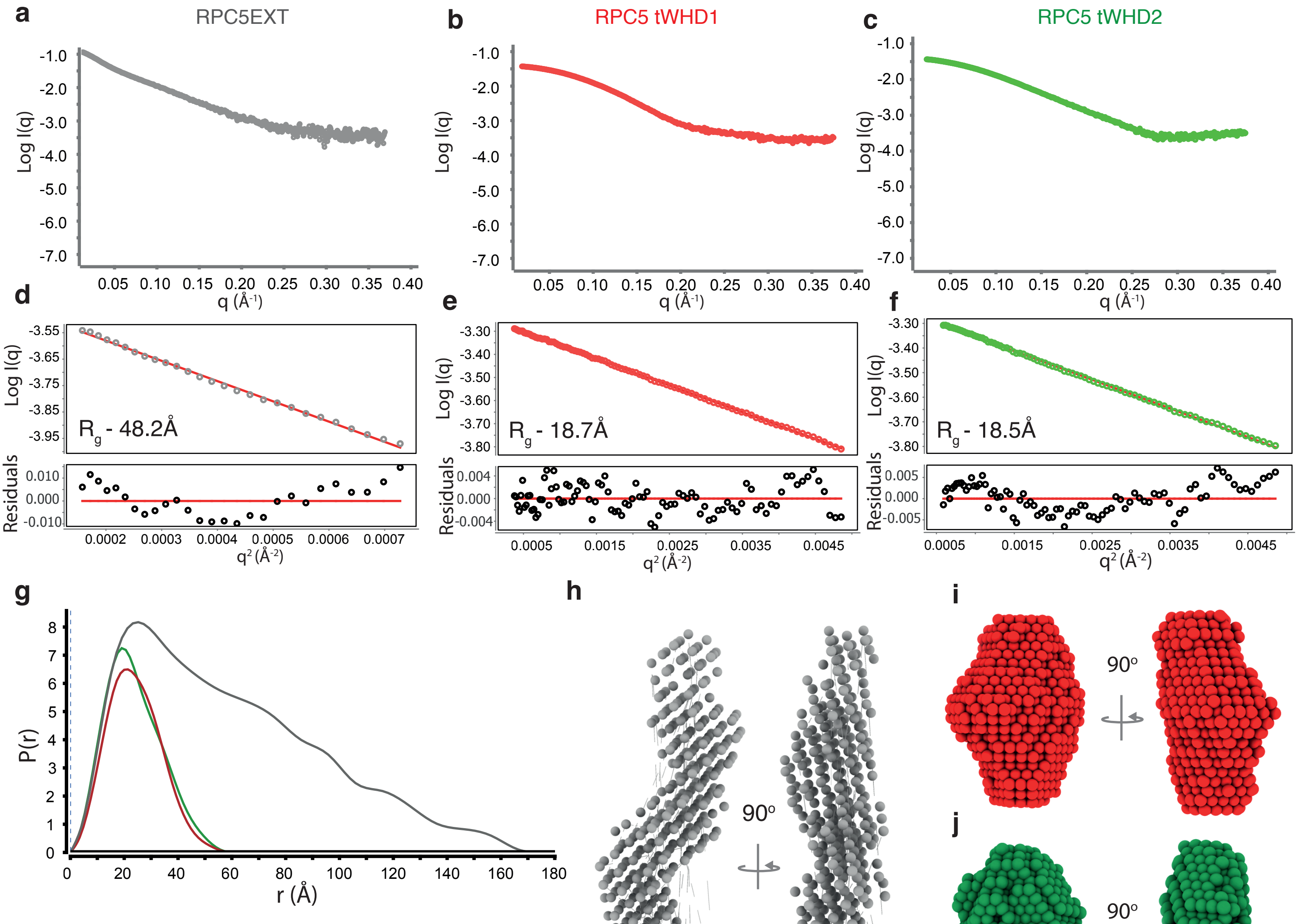

Construct	D_{max}(Å)
RPC5EXT	175
RPC5 tWHD1	61
RPC5 tWHD2	61

Supplementary Figure 8 - SAXS analysis of the RPC5 C-terminus. (a,b,c) One-dimensional scatter curve recorded for the (a) RPC5EXT, (b) RPC5 tWHD1 and (c) RPC5 tWHD2. (d,e,f) Guinier fitting for the recorded scatter curve. Shown is the fitting with the calculated R_g values inset (above) and the residuals for the Guinier fitting across the q-range of the Guinier region (below) for (d) RPC5EXT, (e) RPC5 tWHD1 and (f) RPC5 tWHD2. (g) Overlayed P(r) distruibutions for all constructs (above) with the particle maximum diameter (D_{max}) values determined at the x-intercept (below). Averaged DAMMIN dummy-atom bead models calculated for (h) RPC5EXT, (i) RPC5 tWHD1 and (j) RPC5 tWHD2 constructs.

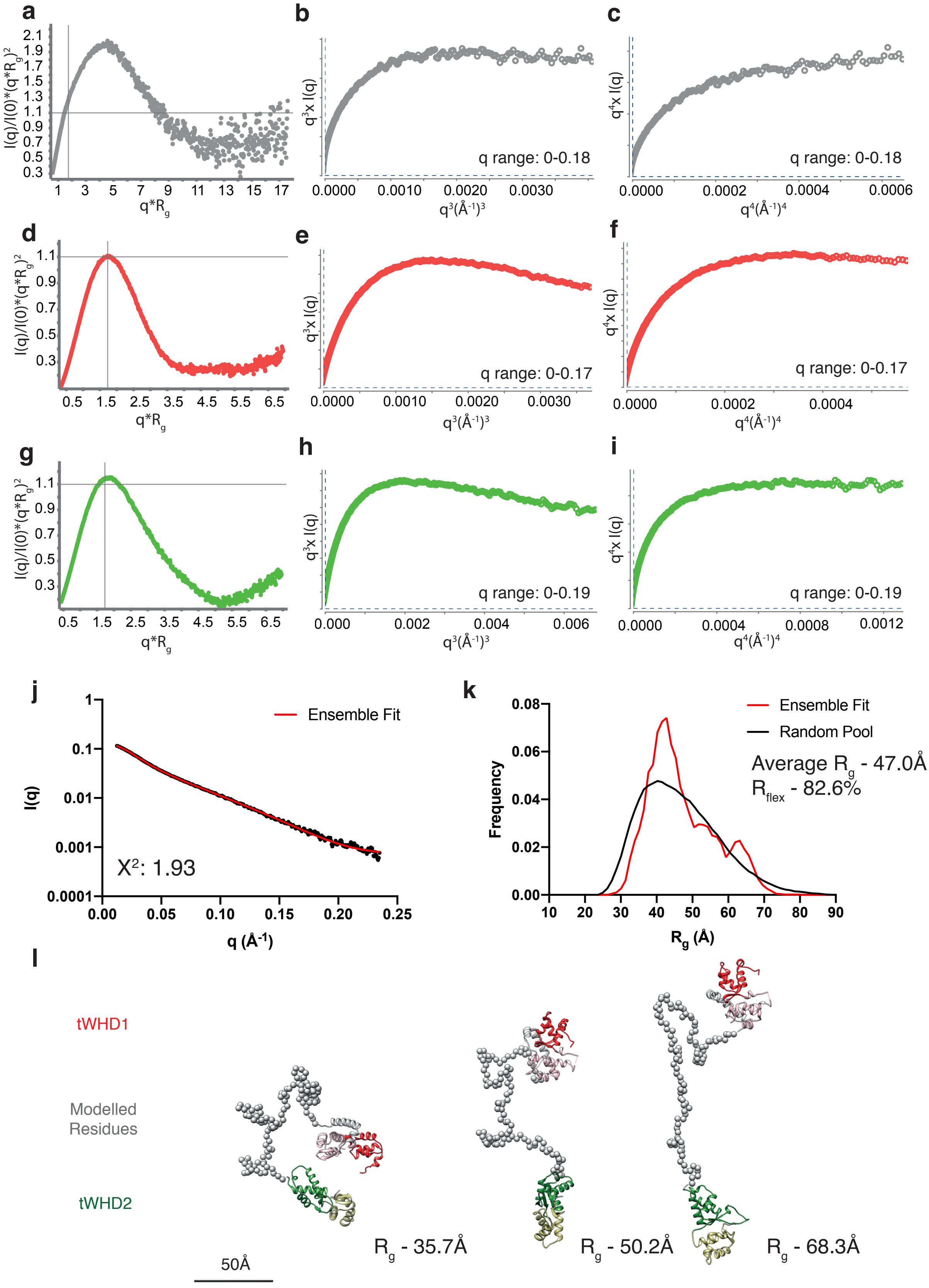

Supplementary Figure 9 - Qualitative assessment of protein flexibility from SAXS data. Calculated R_g normalized kratky plots for (a) RPC5EXT, (d) RPC5 tWHD1 and (g) RPC5 tWHD2. All plots display a bell-shaped profile, indicative of folded protein. Shown also is the intersection at $3^{½}$, 1.1 (crosshairs). A peak observed at this point reports a globular structure, as observed in (d) and (g). Dispersion of the peak away from this point in (a) suggests a more elongated structure. Calculated SIBLYS plots for RPC5EXT (b), RPC5 tWHD1 (e) and RPC5 tWHD2 (h). These are comapred to Porod-Debye plots shown for RPC5EXT (c), RPC5 tWHD1 (f) and RPC5 tWHD2 (i) over the same q-range in each pairwise comparison. The RPC5EXT shows a plateau in the SIBLYS plot before the Porod-Debye, suggestive of a flexible protein. Both RPC5 tWHD1 and RPC5 tWHD2 show a prominent plateau in the Porod-Debye, suggestive of a more rigid body. This suggests the RPC5 C-terminus consists of two rigid bodies with a flexible linker between. (j,k,l) Ensemble optimisation (EOM) analysis of RPC5 C-terminus. Both winged-helix crystal structures were defined as rigid bodies, with EOM modelling the additional 115 residue linker in the RPC5 sequence as dummy atoms. (j) Comparison of the theoretical scattering of the selected ensemble to the experimental SAXS curve for RPC5EXT, with inset the reported fit X^2 value of 1.93 over the q range $0<q<0.2Å^{-1}$. (k) Comparison of the R_g distribution of the random pool of 10000 models (black) to the selected ensemble (red). The resulting ensemble reported an average R_g of 47.0Å and an R_{flex} of 82%. The selected ensemble displayed a wide dispersion of R_g values, equivalent to the range of the random pool, suggesting together with the high R_{flex} value a flexible structure. (l) Selected models from the fitted ensemble with the reported R_g for each showing a range of sizes modelled by the EOM analysis.

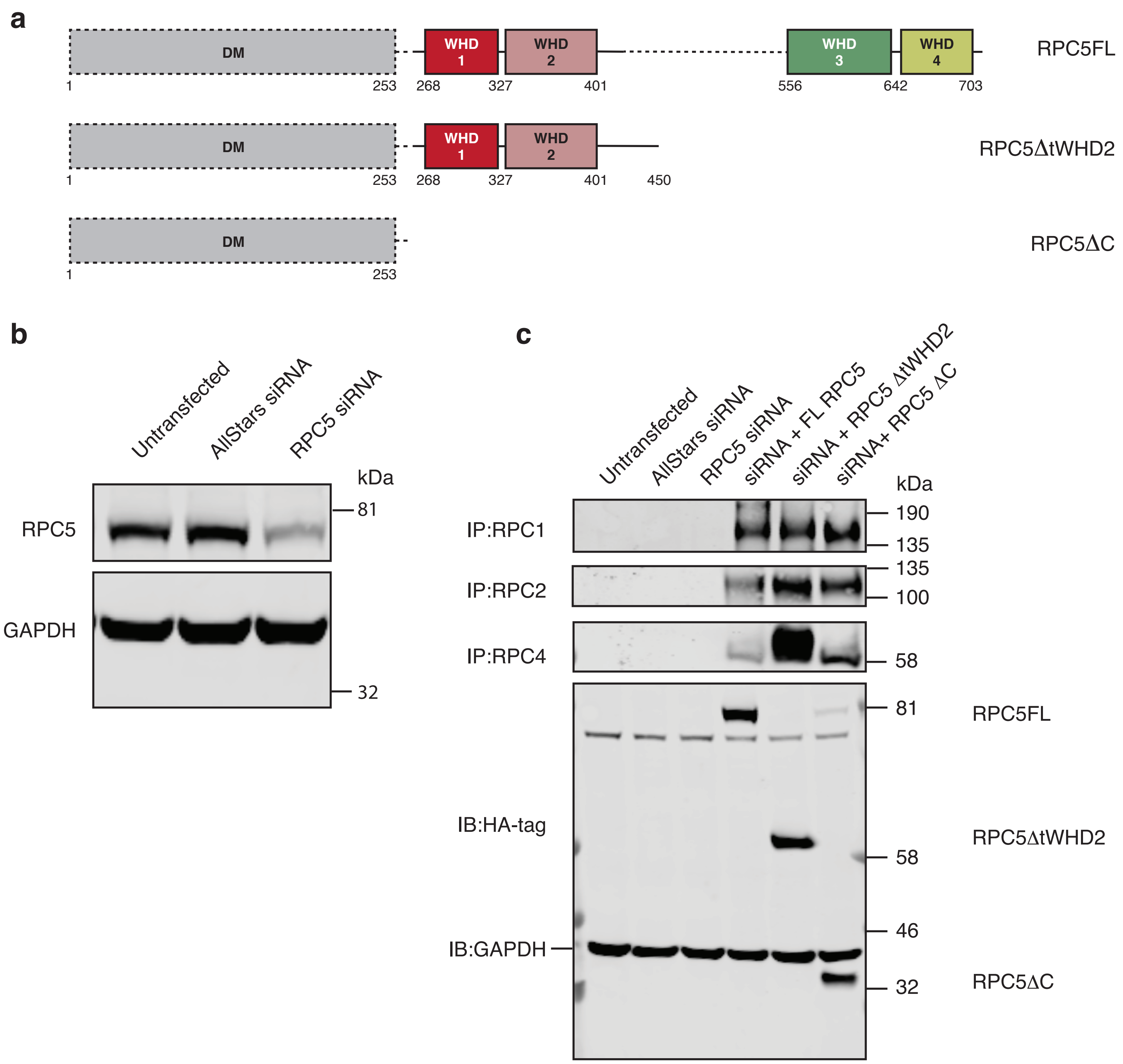

Supplementary Figure 10 - The C-terminus of RPC5 is not required for association with the polymerase core (a) Schematic of N-terminally, HA-tagged DNA constructs that were employed, constituting either amino acids 1-708 RPC5 Full Length (FL), amino acids 1-450 RPC5 (ΔtWHD2) or amino acids 1-253 (ΔC). (b) Western blot confirmation of RPC5 knockdown in HEK293T cells prior to construct rescue. Shown is a representative blot from three independent experiments. (c) Endogenous RPC5 was knocked down using siRNA before either FL, ΔtWHD2 or ΔC HA-tagged RPC5 constructs were transfected. Magnetic HA-beads were used to pull down components of the RNA Pol III complex (RPC1, 2 and 4). From this it was concluded that truncation of RPC5 does not inhibit Pol III complex assembly. Shown is a representative image of three separate repeats. All source data are provided as a Source Data file.

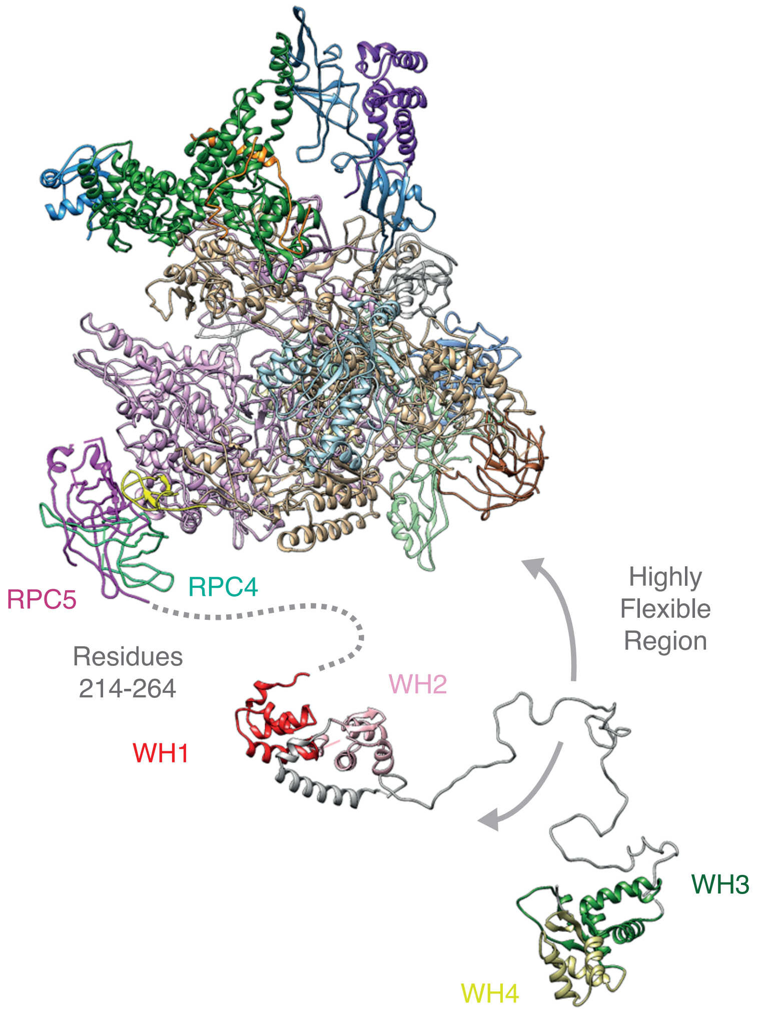

Supplementary Figure 11 - Superimposition of human RNA polymerase III structure and modelled RPC5 C-terminus, distinct regions of RPC5 are labelled.
