# Supplementary material for: Structure of human RNA polymerase III
Source: Nat Commun. 2020 Dec 17;11:6409. doi: 10.1038/s41467-020-20262-5 (PMC7747717; doi:10.1038/s41467-020-20262-5)
Supplement: Supplementary file 3 — Description of Additional Supplementary Files [file 41467_2020_20262_MOESM3_ESM.pdf]

Description of Additional Supplementary Files

File Name: Supplementary Data 1
Description: Overview of human RNA Polymerase III disease associated mutations and their effects on enzyme structure.
