# Supplementary material for: Structure of human RNA polymerase III
Source: Nat Commun. 2020 Dec 17;11:6409. doi: 10.1038/s41467-020-20262-5 (PMC7747717; doi:10.1038/s41467-020-20262-5)
Supplement: Supplementary file 5 — Reporting Summary [file 41467_2020_20262_MOESM5_ESM.pdf]

nature research

Corresponding author(s): Alessandro Vannini and Christoph Engel

Last updated by author(s): Oct 30, 2020

Reporting Summary

Nature Research wishes to improve the reproducibility of the work that we publish. This form provides structure for consistency and transparency in reporting. For further information on Nature Research policies, see our Editorial Policies and the Editorial Policy Checklist.

Statistics

For all statistical analyses, confirm that the following items are present in the figure legend, table legend, main text, or Methods section.

n/a	Confirmed	
☒	☐	The exact sample size (*n*) for each experimental group/condition, given as a discrete number and unit of measurement
☒	☐	A statement on whether measurements were taken from distinct samples or whether the same sample was measured repeatedly
☒	☐	The statistical test(s) used AND whether they are one- or two-sided<br>*Only common tests should be described solely by name; describe more complex techniques in the Methods section.*
☒	☐	A description of all covariates tested
☒	☐	A description of any assumptions or corrections, such as tests of normality and adjustment for multiple comparisons
☒	☐	A full description of the statistical parameters including central tendency (e.g. means) or other basic estimates (e.g. regression coefficient) AND variation (e.g. standard deviation) or associated estimates of uncertainty (e.g. confidence intervals)
☒	☐	For null hypothesis testing, the test statistic (e.g. *F*, *t*, *r*) with confidence intervals, effect sizes, degrees of freedom and *P* value noted<br>*Give P values as exact values whenever suitable.*
☒	☐	For Bayesian analysis, information on the choice of priors and Markov chain Monte Carlo settings
☒	☐	For hierarchical and complex designs, identification of the appropriate level for tests and full reporting of outcomes
☒	☐	Estimates of effect sizes (e.g. Cohen's *d*, Pearson's *r*), indicating how they were calculated

Our web collection on statistics for biologists contains articles on many of the points above.

Software and code

Policy information about availability of computer code

Data collection	EPU v2.4
Data analysis	cisTEM1.0.0, CTFFIND 4.1.5, Cryosparc v2, Relion 3.1, Motioncor2, COOT 0.8.9.2, PHENIX 1.18.1-3865, UCSF Chimera 1.13.1, MacPymol 1.8.6.0, PHYRE-2 v2.0, Scatter v1.0, ATSAS 3.0.1, EOM v2.1, DAMMIN v5.3, DAMAVER v5.0, CRYSOL v3.0, HKL2Map 0.3.i-beta, Buccaneer 1.6.0, STRIDE (http://webclu.bio.wzw.tum.de/stride/), SHELXC v2013/2, SHELXD v2013/2, SHELXE v2014/4, Zeiss ZEN 3.0 (ZEN lite), Fiji v2.0.0-rc-69/1.52p

For manuscripts utilizing custom algorithms or software that are central to the research but not yet described in published literature, software must be made available to editors and reviewers. We strongly encourage code deposition in a community repository (e.g. GitHub). See the Nature Research guidelines for submitting code & software for further information.

Data

Policy information about availability of data

All manuscripts must include a data availability statement. This statement should provide the following information, where applicable:

- Accession codes, unique identifiers, or web links for publicly available datasets
- A list of figures that have associated raw data
- A description of any restrictions on data availability

The electron density reconstructions and final model were deposited with the Electron Microscopy Data Base under accession code nos. EMD-11904, and with the Protein Data Bank (PDB) under accession code 7AST.

The PDB accession numbers for the atomic coordinates and structure factors of the RPC5EXT tWHD1 and tWHD2 crystal structures reported in this paper are 7ASU, and 7ASV, respectively.

The 6EU2 and 6EU3 datasets used for human structure determination and structural comparison are available from RCSB PDB database (https://www.rcsb.org/structure/6EU2; https://www.rcsb.org/structure/6EU3).

Field-specific reporting

Please select the one below that is the best fit for your research. If you are not sure, read the appropriate sections before making your selection.

☒ Life sciences ☐ Behavioural & social sciences ☐ Ecological, evolutionary & environmental sciences

For a reference copy of the document with all sections, see nature.com/documents/nr-reporting-summary-flat.pdf

Life sciences study design

All studies must disclose on these points even when the disclosure is negative.

Sample size	No statistical methods were used to predetermine sample size. The total amount of data collected during the experiment was the total data available for analysis in each case.
Data exclusions	No data were excluded initially from the analysis. During cryoEM analysis, picked coordinates which did not correspond to protein particles were discarded after 2D classification. Particles which did not correspond to the complete apo human RNA Pol III enzyme were discarded following global and local 3D classification, giving the final particle set of 25369 particles used for the final refined reconstruction, which represents an averaged 3D representation of these particles.
Replication	Experimental findings were reproduced as 3 indepenedent cryo-EM data sets (1 untiled and 2 tilted at 30 degrees) which were then merged to give the final maximally resolved structure. The resulting structure is an average of 25369 individual complex particles following 2D and 3D classification. For SAXS data, the reulting 1D scatter curve used for structural analysis is the product of many radially averaged scatter profiles acquired during the experiment. DAMMIN bead models presented are averages of 10 separate modelling calculations for each construct. For X-ray crystallography diffraction several datasets were collected at various wavelengths. The IP, RPC5 siRNA knockdown and cycloheximide chase experiments were repeated three times. All purifications were repeated in excess of three times (human Pol III was purified 9 times across two different laboratories as part of this study) and gave reproducible material for analysis. Confocal imaging showing polymerase localization was repeated 4 times. Cell fractionation and small scale purification from each fraction was repeated twice. The in vitro RNA extension assay was repeated 7 times. In all cases, all attempts at replication were successful.
Randomization	N/A to this study as purifications were directed to isolate specific proteins of interest and in structural determination all data is included in a single analysis. Experiments of RPC5EXT structure and stability/complex association with the polymerase were targeted to understand the role of this region, unqiue to higher eukaryotes, in the Pol III perspective and allow insight into the function of this region.
Blinding	N/A for cryoEM, SAXS and X-ray crystallography analysis, as purified samples were prepared to produce standard grids or crystals which were compatible for structural determination using these techniques. Subsequent data collection and analysis is then automated and does not require blinding.

Reporting for specific materials, systems and methods

We require information from authors about some types of materials, experimental systems and methods used in many studies. Here, indicate whether each material, system or method listed is relevant to your study. If you are not sure if a list item applies to your research, read the appropriate section before selecting a response.

Materials & experimental systems

n/a	Involved in the study	
☐	☒	Antibodies
☐	☒	Eukaryotic cell lines
☒	☐	Palaeontology and archaeology
☒	☐	Animals and other organisms
☒	☐	Human research participants
☒	☐	Clinical data
☒	☐	Dual use research of concern

Methods

n/a	Involved in the study	
☒	☐	ChIP-seq
☒	☐	Flow cytometry
☒	☐	MRI-based neuroimaging

Antibodies

Antibodies used	1. POLR3A (Rb, 1:1000, Abcam, ab96328, lot:GR3281075-3)<br>2. POLR3B (Rb, 1:1000, Abcam, ab137030, EPR8719, lot: YJ040910CS)<br>3. POLR3D (Rb, 1:1000, Abcam, ab86786, lot: GR267691-2)<br>4. POLR3E (Rb, 1:1000, Abcam, ab134560, lot: GR276144-8)<br>5. HA-tag (Rb, 1:1000, Abcam, ab9110, lot: 2842113)<br>6. GAPDH (Ms, 1:5000, MERCK, MAB374, 6C5, lot: 2)

7. RPA40 (RPAC1) (Ms, 1:1000, Santa Cruz, sc-374443 (H-6))
8. Anti-Rabbit IgG (H+L) DyLightTM 800 4x PEG conjugate (1:1000, #5151, Cell Signalling Technology, lot: 3)
9. Anti-Mouse IgG (H+L) DyLightTM 680 (1:1000, #5470, Cell Signalling Technology, lot: 3)

Validation

All antibodies were validated by manufacturers for the applications and species used in this study. See manufacturers websites:
1. https://www.abcam.com/polr3a-antibody-ab96328.html;
2. https://www.abcam.com/polr3b-antibody-epr8719-ab137030.html;
3. https://www.abcam.com/polr3d-antibody-ab86786.html;
4. https://www.abcam.com/polr3e-antibody-ab134560.html;
5. https://www.abcam.com/ha-tag-antibody-chip-grade-ab9110.html;
6. https://www.sigmaaldrich.com/catalog/product/mm/mab374?lang=en&region=GB;
7. https://www.scbt.com/p/rpa40-antibody-h-6;
8. https://www.cellsignal.com/products/secondary-antibodies/anti-rabbit-igg-h-l-dylight-800-4x-peg-conjugate/5151?Ntk=Products&Ntt=5151;
9. https://www.cellsignal.co.uk/products/secondary-antibodies/anti-mouse-igg-h-l-dylight-680-conjugate/5470?Ntk=Products&Ntt=5470.

A summary of the manufacturer validation:

1. POLR3A: Reacts with mouse and human. Tested Application: WB, ChIP
Choquet K et al. Leukodystrophy-associated POLR3A mutations down-regulate the RNA polymerase III transcript and important regulatory RNA BC200. J Biol Chem 294:7445-7459 (2019).
Frischknecht L et al. BRAF inhibition sensitizes melanoma cells to a-amanitin via decreased RNA polymerase II assembly. Sci Rep 9:7779 (2019).
Lee YL et al. MAF1 represses CDKN1A through a Pol III-dependent mechanism. Elife 4:e06283 (2015). ChIP, WB ; Human .

2. POLR3B: Reacts with mouse, rat, human. Tested Applications: Flow Cyt, WB

3. POLR3D: Reacts with human. Tested Applications: WB, IP, IHC-P
Van Bortle K et al. Topological organization and dynamic regulation of human tRNA genes during macrophage differentiation. Genome Biol 18:180 (2017).
Wang J et al. Cytoskeletal Filamin A Differentially Modulates RNA Polymerase III Gene Transcription in Transformed Cell Lines. J Biol Chem 291:25239-25246 (2016).

4. POLR3E: Reacts with human, predicted to react with Chimpanzee, Gorilla. Tested Applications: WB, IP

5. HA-Tag: Reacts with: Species independent. Tested applications: ChIP/Chip, IP, ELISA, WB, ICC/IF, ICC, Flow Cyt, ChIP.

6. GAPDH: Reacts with dog, human, mouse, rat, rabbit, fish, cat, pig. Tested Applications: WB, ELISA, IF, IC.

7. RPA40: Mouse monoclonal antibody raised against residues 1-105 of human RPA40 (RPAC1). Reacts with mouse, rat and human sequences. Suitable for WB (dilution range 1:100-1:1000), IP (1-2ug per 100-500ug of total protein, IHC (dilution range 1:50-1:500), IF (dilution range 1:50-1:500) and ELISA (1:30-1:3000).

8. Anti-Rabbit IgG (H+L) Secondary Antibody. Goat antibody, reacts with heavy and light chains of rabbit immunoglobulins, suitable and validated for WB applications.

9. Anti-Mouse IgG (H+L) Secondary Antibody. Goat antibody, reacts with heavy and light chains of mouse immunoglobulins, suitable and validated for WB applications.

Eukaryotic cell lines

Policy information about cell lines

Cell line source(s)

1. HeLa POLR1C-GFP, produced in this study. HeLa strain is of high passage number originating from the CCL-2 strain. It was established in the Cordes Laboratory (Max Planck Institute).
2. HEK293T (from Dr. Sebastian Guettler).

Authentication

1. HeLa POLR1C-GFP - Insertion of GFP into the genomic DNA was confirmed by PCR. HeLa cell type was confirmed by STR typing.
2. HEK293T cells were not authenticated as part of this study.

Mycoplasma contamination

Cell lines tested negative for mycoplasma contamination.

Commonly misidentified lines
(See ICLAC register)

None.
